# Preclinical Mouse Models of Rheumatoid Arthritis: Comparative Insights into Inflammatory Mediators and Immune Cells

**DOI:** 10.3390/ijms27209037

**Published:** 2026-10-12

**Authors:** Bob Arthur Lubamba

**Affiliations:** Inotiv Boulder, 5541 Central Ave. Suite #160, Boulder, CO 80301, USA; bob.lubamba@inotiv.com or lubamba247@gmail.com; Tel.: +1-919-904-9829

**Keywords:** rheumatoid arthritis, mouse model, CIA, CAIA, human TNF transgenic, SKG

## Abstract

Rheumatoid arthritis (RA) is a chronic autoimmune disease characterized by persistent synovial inflammation, progressive cartilage destruction, and bone erosion. Mouse models are essential for investigating RA pathogenesis, defining immune-cell and cytokine networks, and evaluating potential therapies. Common models include collagen-induced arthritis (CIA), collagen antibody-induced arthritis (CAIA), human TNF transgenic (hTNF-Tg) arthritis, and SKG arthritis, each representing distinct inflammatory mechanisms. CIA involves adaptive and innate immune cells, including CD4^+^ T cells, B cells, dendritic cells, macrophages, and neutrophils, with prominent roles for TNF-α, IL-1β, IL-6, IL-17A, GM-CSF, and IL-23. CAIA primarily reflects antibody-mediated and innate immune mechanisms involving neutrophils, macrophages, complement, and Fcγ receptors, with contributions from TNF-α, IL-1β, IL-6, GM-CSF, CXCL1, and CXCL2. The hTNF-Tg model highlights chronic TNF-driven inflammation involving macrophages and neutrophils and downstream inflammatory mediators such as IL-1β, IL-6, CXCL1, and CCL2. In contrast, the SKG model emphasizes autoreactive CD4^+^ T-cell and Th17 responses, with IL-17A, IL-6, and IL-23 contributing to arthritis following environmental stimulation. Collectively, these models are mechanistically complementary and provide distinct insights into immune activation, cytokine signaling, and joint pathology. However, differences in immune regulation, disease heterogeneity, and inflammatory mechanisms between mice and humans emphasize the importance of careful model selection and interpretation. This review summarizes major mouse models of RA, focusing on their immune-cell and inflammatory mediator networks, therapeutic applications, and translational relevance to human disease.

## 1. Introduction

Rheumatoid arthritis (RA) is a chronic, autoimmune disease characterized by persistent synovial inflammation, progressive cartilage degradation, bone erosion, and irreversible joint damage, ultimately resulting in impaired joint function, reduced mobility, and long-term disability [1,2]. Although substantial advances have been made in understanding RA pathogenesis and developing effective disease-modifying antirheumatic drugs (DMARDs) and biologic therapies, the initiating events that trigger autoimmune activation and sustain chronic disease progression remain incompletely defined [3,4,5]. RA develops through complex interactions among genetic susceptibility, environmental exposures, and dysregulated innate and adaptive immune responses, involving multiple immune-cell populations and inflammatory mediators [5,6,7]. Because the earliest molecular and cellular events preceding clinically apparent disease are difficult to investigate directly in humans, experimental models remain essential for dissecting pathogenic mechanisms, identifying therapeutic targets, and evaluating the efficacy and translational potential of novel interventions [8,9,10].

Animal models have played a fundamental role in advancing RA research and have contributed substantially to the discovery, validation, and optimization of therapeutic strategies [11,12,13]. Mouse models provide a highly controlled and experimentally tractable platform for dissecting the cellular and molecular mechanisms underlying immune activation, inflammatory signaling, synovial pathology, cartilage destruction, and pathological bone remodeling, while also enabling genetic manipulation and systematic evaluation of pharmacological interventions [13,14]. These models are widely used to assess the efficacy, mechanisms of action, dose–response relationships, and potential limitations of conventional DMARDs, biologic agents, and emerging targeted therapies [15,16,17,18].

Researchers have developed a broad spectrum of mouse models to reproduce distinct immunological and pathological components of human RA, including spontaneous autoimmune, antigen-induced, antibody-mediated, cytokine-driven, and genetically modified models [19]. These models differ substantially in disease-induction mechanisms, dependence on innate versus adaptive immunity, cytokine profiles, clinical course, and patterns of joint pathology. Consequently, no single model fully recapitulates the complex, heterogeneous nature of human RA [20]. Murine and human arthritis also differ in disease kinetics, immune regulation, joint anatomy, and skeletal remodeling [8]. For example, experimental inflammatory arthritis in rodents can be accompanied by both bone erosion and pathological periosteal or endosteal bone formation. In contrast, human RA is predominantly characterized by pathological bone loss and erosive joint destruction [1,21]. These interspecies differences emphasize the importance of selecting experimental models based on the specific biological or therapeutic question being investigated and interpreting preclinical findings within their appropriate translational context.

Among the established experimental systems, collagen-induced arthritis (CIA), collagen antibody-induced arthritis (CAIA), human TNF transgenic (hTNF-Tg) arthritis, and SKG arthritis represent complementary models for investigating distinct components of RA pathogenesis and therapeutic response [6,11,20]. CIA is primarily useful for studying antigen-specific adaptive immunity and interactions among T-cell, B-cell, and innate inflammatory responses during autoimmune arthritis [9,10]. In contrast, CAIA is induced by the passive transfer of arthritogenic antibodies and therefore provides a model in which inflammatory effector mechanisms can be investigated independently of the initial development of adaptive autoimmunity [12,22]. The hTNF-Tg model is driven by sustained overexpression of human TNF-α and is particularly valuable for studying cytokine-dependent synovial inflammation and evaluating therapies targeting the TNF pathway [15,18,23]. SKG mice, which carry a point mutation in the Zap70 gene affecting T-cell selection and signaling, develop autoimmune arthritis through dysregulated T-cell responses and are therefore particularly informative for investigating T-cell-mediated autoimmune mechanisms and environmental triggers of disease [19,24] (Figure 1).

## 2. Experimental Rheumatoid Arthritis Mouse Models

Murine models have been fundamental to elucidating the immunopathology of chronic inflammatory arthritis and to preclinical evaluation of disease-modifying therapies [7,8]. No single model reproduces the complexity and heterogeneity of human RA; instead, individual models capture selected features of autoimmune initiation, antibody-mediated inflammation, cytokine-driven disease, or genetically determined immune dysregulation [11]. Together, CIA, CAIA, hTNF-Tg, and SKG arthritis provide complementary experimental systems. CIA emphasizes antigen-specific adaptive immunity; CAIA isolates antibody-mediated effector inflammation; hTNF-Tg captures sustained cytokine-driven disease; and SKG links defective immune tolerance to environmental triggers and Th17-associated inflammation. Together, they can help distinguish mechanism-specific activity from nonspecific anti-inflammatory effects. At the same time, human translational studies remain essential for assessing relevance to patient disease (Table 1).

### 2.1. Mouse Collagen-Induced Arthritis

Among the RA models, CIA remains one of the most extensively characterized and widely used experimental systems for investigating RA pathogenesis, identifying therapeutic targets, and evaluating immune-modulating interventions [9,10]. Since its initial description, the CIA model has substantially advanced understanding of interactions between innate and adaptive immune responses, inflammatory cytokine networks, autoantibody generation, and mechanisms responsible for cartilage and bone destruction [25].

CIA’s translational relevance is underscored by its role in developing and validating biologic therapies that target key inflammatory pathways. Studies using CIA and related arthritis models have provided important evidence supporting the pathogenic role of TNF-α, a proinflammatory cytokine produced by macrophages, T cells, and other immune cells. Persistent TNF-α signaling promotes synovial inflammation, leukocyte recruitment, fibroblast activation, osteoclast differentiation, and progressive joint destruction. The successful clinical application of TNF inhibitors and other targeted immunotherapies has demonstrated the value of experimental arthritis models in advancing therapeutic discovery and improving treatment strategies for patients with RA. CIA recapitulates multiple pathological and immunological features observed in human RA, including synovial inflammation, immune cell infiltration, autoantibody production, pannus formation, and progressive cartilage and bone erosion [26].

CIA is induced by immunization and boosted with type II collagen (CII), the major structural protein of articular cartilage, in the presence of Freund’s complete adjuvant or Freund’s incomplete adjuvant. CII acts as a cartilage-associated autoantigen that activates collagen-specific T- and B-cell responses, leading to anti-collagen antibody production and immune-complex-mediated inflammation. Subsequent activation of complement pathways, Fc gamma receptor (FcγR)-dependent signaling, and inflammatory cytokine production promotes the recruitment and activation of innate immune cells, particularly macrophages and neutrophils, leading to synovial inflammation and joint destruction [25].

Compared with other antigen-specific arthritis models, CIA offers several advantages, including a relatively predictable disease course, reproducible clinical manifestations, and the ability to investigate both the initiation and progression of autoimmune arthritis. In addition, CIA provides a robust platform for evaluating the efficacy of anti-inflammatory agents, biologic therapies, and small-molecule inhibitors before clinical translation.

Despite its extensive utility, several limitations should be considered when interpreting findings from CIA studies. Disease susceptibility and severity vary considerably across mouse strains, major histocompatibility complex (MHC) haplotypes, sex, and experimental conditions. Furthermore, although CIA shares many pathological characteristics with human RA, differences remain in immune regulation, antigen specificity, disease heterogeneity, and the genetic factors that contribute to disease susceptibility. Therefore, the results obtained from CIA studies should be integrated with findings from complementary arthritis models and human clinical investigations.

Overall, the CIA model remains a cornerstone of preclinical RA research because it closely recapitulates several key features of autoimmune inflammatory arthritis and enables mechanistic investigation of disease pathways and evaluation of novel therapeutic strategies.

### 2.2. Mouse Collagen Antibody-Induced Arthritis

CAIA is a widely used murine model for investigating the effector mechanisms underlying inflammatory arthritis and evaluating therapeutic strategies targeting established inflammatory pathways [13,27,28]. Unlike CIA, which requires active immunization with CII and the development of collagen-specific adaptive immune responses, CAIA is induced by the passive transfer of monoclonal antibodies directed against CII, typically followed by an inflammatory stimulus such as lipopolysaccharide (LPS) [13,27]. This approach bypasses the priming phase of autoimmune activation and enables direct investigation of downstream inflammatory mechanisms that contribute to synovial inflammation and joint destruction [27,29]. Consequently, CAIA is particularly useful for studying the effector phase of arthritis and for evaluating anti-inflammatory therapies that act downstream of autoantibody production [13,28].

Following administration of anti-CII antibodies, immune complexes form on cartilage-associated CII and within synovial tissues, promoting complement activation and Fc gamma receptor (FcγR)-dependent signaling [27,28,29]. These processes recruit and activate innate immune effector cells, particularly neutrophils, monocytes/macrophages, and other Fc receptor-bearing myeloid cells, resulting in the production of inflammatory cytokines, chemokines, and tissue-degrading mediators [27,28]. Complement activation and FcγR engagement are critical components of antibody-mediated arthritis, and experimental evidence shows that disrupting Fc receptor signaling markedly reduces disease severity [27]. Histopathologically, CAIA reproduces several important features of inflammatory arthritis, including synovial inflammation, inflammatory-cell infiltration, pannus formation, cartilage degradation, and bone erosion [13,27,30].

Antibody-dependent inflammatory mechanisms primarily mediate the molecular basis of CAIA. Commercially available antibody cocktails generally contain multiple monoclonal antibodies that recognize distinct epitopes within CII, increasing the likelihood of effective antibody binding to cartilage and formation of pathogenic immune complexes [25,28]. The arthritogenic potential of individual anti-CII antibodies depends on factors including epitope specificity, antibody isotype, antigen affinity, and complement activation capacity [25]. After deposition of antibody–CII complexes, complement activation and FcγR engagement amplify local inflammation and promote myeloid cell recruitment and activation [25,27,28,29]. These pathways are relevant to human RA, in which autoantibody-containing immune complexes can contribute to synovial inflammation through Fc receptor- and complement-dependent mechanisms [31].

A major advantage of the CAIA model is that disease induction is largely independent of MHC restriction and does not require the priming of collagen-specific T or B cells [28]. Thus, CAIA is particularly valuable for dissecting innate immune mechanisms involving FcγR signaling, complement activation, neutrophil recruitment, macrophage activation, and cytokine-mediated inflammation [27,29]. The rapid and relatively synchronized onset of arthritis represents another important advantage, making CAIA suitable for therapeutic efficacy studies, particularly investigations of compounds targeting inflammatory mediators involved in the established effector phase of arthritis [13,27]. In contrast to CIA, which generally requires several weeks after immunization for disease development, CAIA can produce prominent clinical arthritis within days after antibody administration and LPS challenge [30].

The C57BL/6 mouse strain is frequently used in CAIA studies because its genetic background is compatible with a large collection of genetically modified mouse lines, including knockout and transgenic strains [28,30]. This characteristic enables investigators to examine the contribution of specific genes, receptors, signaling pathways, cytokines, complement components, and myeloid-cell populations to arthritis development. C57BL/6 mice can develop robust antibody-mediated inflammatory arthritis following appropriate CAIA induction, although disease severity may vary according to antibody cocktail composition and dose, LPS administration, mouse age and sex, genetic background, microbiota, and other experimental conditions [28]. Therefore, optimization of antibody and LPS dosing may be necessary to achieve reproducible disease severity across experimental studies.

Despite its utility, CAIA has important limitations. Because disease is induced by passive transfer of preformed anti-CII antibodies, the model does not reproduce the early autoimmune events that characterize human RA, including breakdown of immune tolerance, generation and maturation of autoantibodies, antigen presentation, and development of antigen-specific lymphocyte responses [27,28,30]. Although T and B cells can modulate disease severity during the effector phase, they are not required for initial CAIA induction [28]. Therefore, CAIA should be considered primarily a model of the antibody-driven inflammatory effector phase of arthritis rather than a comprehensive model of RA initiation and chronic autoimmune disease development [27]. Nevertheless, its rapid onset, reproducibility, mechanistic focus, and suitability for genetically modified mice make CAIA an important complementary model for defining inflammatory pathways and evaluating anti-arthritic therapeutic strategies [13,30].

### 2.3. Human Tumor Necrosis Factor Transgenic Mouse Model

The hTNF-Tg mouse is a well-established model of chronic TNF-driven inflammatory arthritis and has provided important insights into the molecular mechanisms underlying persistent synovial inflammation and progressive joint destruction in RA [18,32,33]. Keffer et al. originally developed the model and showed that mice carrying a modified human TNF-α transgene developed predictable chronic inflammatory polyarthritis, and the neutralization of human TNF-α prevented arthritis development, providing early experimental evidence for a central pathogenic role of TNF-α [18]. Unlike inducible models such as CIA and CAIA, hTNF-Tg mice develop arthritis spontaneously due to constitutive or dysregulated expression of human TNF-α, without the need for collagen immunization or exogenous inflammatory stimulation [18,34].

The pathological manifestations observed in hTNF-Tg mice reproduce several important features of human RA, including chronic synovitis, synovial hyperplasia, inflammatory-cell infiltration, pannus formation, cartilage degradation, progressive bone erosion, and wrist deformities [18,33,34,35]. Butler et al. demonstrated that hTNF-Tg mice develop severe erosive arthritis accompanied by alterations in the cytokine environment and cellular composition of inflamed joints [33]. Persistent TNF-α signaling activates multiple cellular populations within the joint, including macrophages, synovial fibroblasts, endothelial cells, and osteoclast-lineage cells. These responses promote the production of additional inflammatory mediators and matrix-degrading enzymes, thereby sustaining synovial inflammation, extracellular-matrix degradation, angiogenesis, and pathological bone remodeling [33,36,37]. TNF-driven activation of osteoclast pathways is particularly important for the erosive phenotype, as studies in hTNF-Tg mice have demonstrated that inhibition or depletion of osteoclasts markedly reduces TNF-mediated bone destruction [36,37].

A major strength of the hTNF-Tg model is its ability to reproduce chronic inflammation driven by a cytokine pathway that is directly relevant to human RA. TNF-α occupies a central position within the inflammatory network of RA and contributes to synovial hyperplasia, leukocyte recruitment, fibroblast activation, osteoclastogenesis, angiogenesis, and structural joint damage [1,34]. Consequently, hTNF-Tg mice have been extensively used to investigate TNF-dependent pathogenic mechanisms and to evaluate therapeutic strategies targeting TNF signaling and downstream inflammatory pathways. Importantly, the original studies demonstrated that anti-human TNF antibodies could prevent arthritis in hTNF-Tg mice, providing an early preclinical demonstration that TNF neutralization could effectively suppress TNF-driven joint inflammation [18]. Subsequent studies further showed that anti-TNF treatment reduces inflammatory and structural manifestations of disease and can restore aspects of lymphatic function in arthritic hTNF-Tg mice [38]. These findings contributed to the broader experimental rationale supporting TNF blockade as a therapeutic strategy in RA.

Unlike CIA, arthritis in hTNF-Tg mice is initiated primarily by persistent cytokine overexpression and downstream inflammatory signaling. This characteristic makes the model particularly useful for investigating mechanisms occurring downstream of TNF activation, including macrophage activation, synovial fibroblast responses, angiogenesis, osteoclastogenesis, lymphatic dysfunction, and tissue remodeling [33,38,39]. For example, macrophages are an important cellular population in the inflamed synovium of hTNF-Tg mice, and TNF-dependent activation of osteoclast precursors contributes substantially to pathological bone resorption [36,40]. TNF signaling also promotes angiogenic and lymphangiogenic responses within inflamed joints, further demonstrating the model’s utility for studying interactions among inflammatory cytokines, vascular responses, and tissue remodeling [39].

However, the hTNF-Tg model has important limitations. Because disease initiation results from artificial and sustained overexpression of TNF-α rather than spontaneous loss of tolerance to joint-associated antigens, the model does not reproduce the initiating autoimmune events characteristic of human RA, including the development of rheumatoid factor, anti-citrullinated protein antibodies, and antigen-specific autoreactive lymphocyte responses [34]. Therefore, hTNF-Tg mice are particularly informative for studying the effector phase of chronic inflammatory arthritis and TNF-dependent tissue damage rather than the initiation of systemic autoimmunity. This distinction matters when interpreting therapeutic studies and selecting complementary models to investigate adaptive immune mechanisms.

The genetic background of hTNF-Tg mice can influence disease severity and kinetics. Researchers have generated and maintained several TNF-Tg lines on different genetic backgrounds, including C57BL/6, DBA/1, FVB, and mixed backgrounds [33]. For example, TNF-Tg line 3647 has been extensively crossed onto the C57BL/6J background. It is widely used for mechanistic studies of TNF-driven inflammatory arthritis [41]. In contrast, other transgenic lines, such as TgTC, were generated on an FVB background and also developed progressive erosive polyarthritis that responded to anti-human TNF therapy [35]. Thus, report the specific transgenic line and genetic background when describing experimental findings because they can affect disease onset, severity, and therapeutic responsiveness.

Disease progression in hTNF-Tg mice can be evaluated by longitudinal clinical scoring of paw swelling and joint inflammation, together with histopathological assessment of synovitis, pannus formation, cartilage damage, and bone erosion. Imaging approaches such as radiography and micro-computed tomography can provide complementary measurements of structural joint destruction. At the same time, molecular and cellular analyses can be used to characterize TNF-dependent signaling pathways and immune-cell responses [35,37,40]. The disease’s relatively predictable, progressive course makes hTNF-Tg mice particularly valuable for evaluating biologic agents, small-molecule inhibitors, and therapeutic approaches targeting TNF-dependent inflammatory and tissue-destructive pathways.

Overall, the hTNF-Tg model provides a powerful experimental platform for investigating chronic inflammatory arthritis, particularly mechanisms associated with excessive TNF-α signaling. Its spontaneous and progressive phenotype, reproducible joint pathology, and responsiveness to TNF-targeted interventions make it highly valuable for translational arthritis research [18,34]. Nevertheless, because it primarily models cytokine-driven effector mechanisms rather than the initiation of systemic autoimmunity, it is best used alongside autoimmune models such as CIA, CAIA, and SKG arthritis to provide a more comprehensive understanding of RA pathogenesis and therapeutic responses.

### 2.4. Zymosan-Induced Arthritis in the SKG Mouse Model

The SKG mouse model is a unique genetically predisposed model of autoimmune arthritis that highlights the critical interplay between genetic susceptibility and environmental triggers in RA development. Unlike inducible models that rely on immunization with defined antigens, SKG mice carry a spontaneous hypomorphic point mutation in the gene encoding the T-cell receptor (TCR)-associated signaling molecule ζ-chain-associated protein kinase 70 (ZAP-70). The canonical SKG mutation, ZAP-70 (^W163C), alters TCR signaling thresholds during thymic selection, allowing highly autoreactive CD4^+^ T cells that would normally undergo negative selection to escape into the peripheral immune compartment [24]. These autoreactive T cells provide the genetic substrate for autoimmune arthritis, but environmental stimulation is required for efficient disease development under specific-pathogen-free (SPF) conditions [24].

Administration of zymosan, a yeast cell-wall preparation enriched in β-glucans derived from Saccharomyces cerevisiae, provides a potent innate immune stimulus that reliably induces arthritis in SKG mice maintained under SPF conditions [24]. Zymosan and other fungal β-glucans activate innate immune pathways primarily through pattern-recognition receptors, particularly C-type lectin domain family 7 member A (CLEC7A; Dectin-1), with Toll-like receptor 2 (TLR2) also contributing to recognition and downstream inflammatory signaling [42]. Activation of these pathways promotes spleen tyrosine kinase (Syk)-dependent signaling and downstream activation of nuclear factor-κB (NF-κB) and other inflammatory pathways, resulting in production of cytokines and chemokines that promote the differentiation and expansion of pathogenic T-cell responses [43].

This interaction between innate immune activation and adaptive immune dysregulation closely resembles an important feature of human RA, in which environmental factors may influence the transition from genetic susceptibility to clinically apparent autoimmune disease. The SKG model therefore provides a particularly useful experimental system for investigating how microbial or environmental signals activate pre-existing autoreactive T-cell populations and promote chronic inflammatory arthritis [24,44].

Following a single intraperitoneal injection of zymosan, SKG mice typically develop symmetrical polyarthritis involving peripheral joints, including the ankles, wrists, and digits. Disease generally becomes clinically apparent about 2–3 weeks after β-glucan or zymosan administration. However, onset and severity can vary by dose and preparation of the inducing agent, mouse age and sex, genetic background, microbiota, and housing conditions [45,46]. Histopathological examination demonstrates characteristic inflammatory arthritis, including synovial hyperplasia, inflammatory-cell infiltration, pannus formation, cartilage destruction, and erosive bone changes [24,46].

The SKG model offers several important advantages for RA research. First, it provides a mechanistic framework for investigating how environmental microbial components can initiate autoimmune inflammation in genetically susceptible hosts. Second, it permits investigation of the interaction between innate immune activation, autoreactive CD4^+^ T cells, and pathogenic Th17-cell differentiation. Third, the model is particularly useful for evaluating therapeutic strategies directed toward inflammatory cytokine networks and immune-signaling pathways implicated in RA, including the IL-23/IL-17 axis, IL-6, TNF-α, and JAK-dependent signaling pathways [44]. The model has also been useful for examining IL-23’s contribution to disease development, as IL-23 blockade can attenuate arthritis and related inflammatory manifestations in β-glucan-treated SKG mice [47].

Despite these advantages, several limitations should be considered. Disease severity and reproducibility can be influenced by zymosan source and preparation, dose, mouse age and sex, genetic background, microbiota composition, and housing conditions [46]. Importantly, the strong innate immune stimulation produced by systemic β-glucan exposure does not fully reproduce the heterogeneous environmental exposures or disease initiation processes that occur in human RA. Furthermore, although the SKG model strongly emphasizes Th17/IL-17-mediated inflammation, human RA is immunologically heterogeneous. It involves multiple interacting immune-cell populations and cytokine networks. Therefore, interpret findings from SKG mice alongside results from complementary CIA, CAIA, hTNF-Tg models, and human studies [44,48].

Overall, the zymosan-induced SKG model is a valuable experimental system for studying autoimmune arthritis driven by the interaction between genetic susceptibility and environmental innate immune stimulation. The combination of defective thymic T-cell selection caused by the ZAP-70 mutation and subsequent microbial β-glucan-mediated innate immune activation generates a disease phenotype dominated by autoreactive CD4^+^ T cells and pathogenic Th17/IL-17 responses. This distinctive biology makes the SKG model particularly valuable for investigating the initiation of autoimmunity, mechanisms linking environmental triggers to chronic inflammation, the IL-23/IL-17 cytokine network, and therapeutic strategies targeting immune-cell and inflammatory pathways relevant to RA [24,44,47].

## 3. Immune Cells Involved in CIA, CAIA, hTNF and SKG

The immune-cell populations involved in experimental arthritis vary considerably among the CIA, CAIA, hTNF-Tg, and SKG models, reflecting fundamental differences in disease initiation, antigen specificity, and the relative contribution of adaptive versus innate immune mechanisms. Although these models share several downstream effector cells, including macrophages, neutrophils, monocytes, and osteoclasts, the upstream immune events that recruit and activate these cells differ substantially. CIA involves coordinated activation of antigen-specific CD4^+^ T- and B-cell responses against type II collagen, followed by production of arthritogenic anti-collagen antibodies and recruitment of innate effector cells, including macrophages and neutrophils, into the joints [6,49,50]. CAIA, in contrast, largely bypasses the requirement for antigen-specific adaptive immune priming by directly transferring pathogenic anti-collagen antibodies, thereby activating Fcγ receptor- and complement-dependent inflammatory pathways and promoting recruitment of innate effector cells [29,51,52]. The hTNF-Tg model differs because constitutive overexpression of human TNF-α drives arthritis, resulting in sustained TNF-dependent activation of inflammatory pathways and progressive synovial inflammation, cartilage destruction, and osteoclast-mediated bone resorption [17,18,35]. In this model, myeloid cells and other TNF-responsive stromal and joint-resident cells amplify and sustain inflammation. At the same time, osteoclasts are particularly important for TNF-mediated bone destruction [53]. Conversely, SKG arthritis is initiated by a hypomorphic mutation in the T-cell receptor signaling molecule ZAP-70, which alters thymic selection and permits autoreactive CD4^+^ T cells to escape negative selection. Following appropriate environmental stimulation, these autoreactive T cells differentiate toward pathogenic Th17 cells and drive chronic inflammatory arthritis through IL-17-dependent mechanisms [24,54]. Collectively, these differences in immune-cell recruitment and activation determine the suitability of each model for investigating distinct aspects of RA pathogenesis and therapeutic intervention, including antigen-specific adaptive immunity in CIA, antibody- and innate effector mechanisms in CAIA, TNF-dependent myeloid and stromal inflammation in hTNF-Tg arthritis, and autoreactive T-cell/Th17-driven disease in SKG mice [6,11] (Table 2).

### 3.1. Mouse Collagen-Induced Arthritis

CIA is a complex autoimmune arthritis model induced by immunization with CII. After immunization, antigen-presenting cells, particularly dendritic cells (DCs), process and present collagen-derived peptides to naïve CD4^+^ T cells in secondary lymphoid organs. This promotes activation and differentiation of collagen-reactive T cells and establishes the antigen-specific adaptive immune response required for disease development [55,56]. CD4^+^ T cells are therefore important upstream regulators of CIA, with Th1 and Th17 populations contributing to inflammatory amplification. Th17 cells are particularly important because IL-17 promotes chemokine production, neutrophil recruitment, activation of synovial fibroblasts, and osteoclastogenic pathways, thereby linking adaptive immunity to tissue destruction [57,58]. Th17 cells can also contribute directly to arthritic bone destruction through interactions with osteoclastogenic pathways [58]. Activated CD4^+^ T cells can also stimulate B-cell differentiation and antibody production, creating an interconnected T-cell–B-cell axis characteristic of the CIA model [59,60].

B cells have a particularly important role in CIA because they generate antibodies against CII. Following T-cell-dependent B-cell activation, B cells differentiate into antibody-secreting plasma cells and produce collagen-specific IgG antibodies. These antibodies recognize CII within articular tissues and form immune complexes that activate complement and Fcγ receptors on innate immune cells. Consequently, the humoral response initiated during CIA is not simply an indicator of disease development but contributes directly to the transition from systemic autoimmunity to localized joint inflammation [27,61]. Distinct Fcγ receptor pathways have been shown to regulate CIA induction and severity, providing evidence that immune-complex-mediated activation of innate immune cells is a major component of disease pathogenesis [61]. This antibody-dependent component also explains why serum or purified arthritogenic anti-CII antibodies can reproduce an inflammatory arthritis phenotype in antibody-transfer models [27].

DCs contribute to CIA at multiple stages. During initiation, they act as professional antigen-presenting cells that activate collagen-specific T cells. Within inflamed joints, DCs can maintain and amplify local inflammation through interactions with T cells and production of inflammatory mediators. Thus, DCs provide an important connection between antigen recognition and adaptive immune activation [55,56].

Once collagen-specific antibodies and immune complexes accumulate in the joint, innate immune cells become increasingly important. Macrophages are major effector cells in established CIA and respond to immune complexes, complement-derived signals, and inflammatory cytokines. Activated macrophages produce TNF-α, IL-1β, IL-6, chemokines, reactive oxygen species, and matrix-degrading enzymes, thereby promoting synovial inflammation, cartilage degradation, and osteoclast differentiation [27,55]. Neutrophils are recruited into the joint in response to chemokines and complement-derived anaphylatoxins and contribute to acute inflammatory pathology by releasing reactive oxygen species, proteases, neutrophil extracellular traps, and other tissue-damaging mediators [62]. The cellular composition of CIA therefore shifts from an antigen-specific adaptive response to a mixed adaptive-innate inflammatory infiltrate. Histologically, macrophages, granulocytes, DCs, and activated synovial cells become prominent within the inflamed pannus, whereas T cells and B cells continue to provide upstream immunological signals [55,56]. Osteoclasts subsequently mediate much of the bone erosion by responding to inflammatory cytokines and osteoclastogenic signals such as RANKL. IL-17 can further promote osteoclastogenesis and bone destruction, providing an important mechanistic link between Th17-mediated inflammation and skeletal damage in CIA [58,63].

Thus, CIA is best viewed as a multistage model in which CD4^+^ T cells and B cells initiate antigen-specific immunity. In contrast, macrophages, neutrophils, and osteoclasts execute much of the downstream inflammatory and destructive response. The close interaction between adaptive and innate immune compartments makes CIA particularly valuable for investigating the coordinated immunopathogenesis of RA [9,27,55].

### 3.2. Mouse Collagen Antibody-Induced Arthritis

CAIA bypasses the need to actively generate collagen-specific adaptive immunity by administering preformed pathogenic anti-CII antibodies. Consequently, endogenous B cells and CD4^+^ T cells are not essential for disease initiation. Instead, the pathogenic process begins when transferred anti-CII antibodies bind to cartilage-associated collagen and form immune complexes within the joint. These immune complexes activate complement and engage activating FcγRs expressed on neutrophils, macrophages, monocytes, and other myeloid cells, rapidly initiating local inflammation. FcγR signaling is particularly important because it converts antibody deposition into cellular activation and the release of inflammatory mediators. Experimental studies have demonstrated that FcγR signaling and complement activation are central components of antibody-mediated arthritis. However, B-cell-deficient mice remain susceptible to CAIA, supporting the relative independence of disease induction from endogenous adaptive immune activation [14,64,65].

Neutrophils are among the most prominent effector cells in CAIA and contribute substantially to the rapid onset and severity of joint inflammation. Complement activation generates C3a and C5a, with C5a serving as a potent chemoattractant and activator that promotes neutrophil recruitment to antibody-deposited joints. However, FcγR engagement on neutrophils promotes cellular activation and release of reactive oxygen species, proteases, cytokines, and other inflammatory mediators. Experimental depletion studies have demonstrated that neutrophils are critical for the development and severity of CAIA. At the same time, genetic disruption of complement receptors markedly reduces neutrophil accumulation and disease severity. Similarly, studies of FcγR-associated signaling demonstrate that Syk-dependent signaling in neutrophils is indispensable for antibody-mediated arthritis, highlighting the importance of FcγR-Syk pathways in neutrophil activation [14].

Macrophages and monocytes provide another major effector compartment in CAIA. These cells recognize immune complexes through activating FcγRs and produce TNF-α, IL-1β, chemokines, and other inflammatory mediators that amplify leukocyte recruitment and synovial inflammation. Macrophage-derived inflammatory mediators can also promote osteoclast differentiation and thereby contribute indirectly to cartilage and bone destruction. Studies of antibody-mediated arthritis support the importance of macrophage-derived cytokines by demonstrating pathogenic roles for TNF-α and IL-1β, and broader studies show that macrophage activation is an important component of immune-complex-driven joint inflammation [64,65]. In addition, mast cells can contribute to the early inflammatory response by responding to immune complexes and complement-derived signals, although their relative contribution can vary by experimental model, mouse strain, antibody preparation, and inflammatory stimulus [66,67].

Importantly, CAIA should not be interpreted as completely independent of adaptive immunity during the entire disease course. Although endogenous T- and B-cell responses are largely dispensable for CAIA induction, adaptive immune cells can influence the magnitude and duration of inflammation under some experimental conditions. Nevertheless, the dominant mechanism during the acute effector phase is antibody–FcγR–complement activation followed by recruitment and activation of neutrophils and macrophages. This distinction makes CAIA particularly useful for separating antibody-dependent effector mechanisms from antigen-specific T- and B-cell responses. The model therefore provides an experimental system for dissecting the effector phase of inflammatory arthritis without requiring the preceding antigen-specific immune-priming events characteristic of CIA [14,67].

Overall, CAIA is characterized by a rapid, antibody-driven inflammatory response in which neutrophils and macrophages serve as principal cellular effectors, with complement and FcγR signaling providing critical links between antibody deposition and tissue inflammation. In contrast to CIA, the model minimizes the contribution of antigen presentation, collagen-specific T-cell activation, and endogenous B-cell differentiation during disease induction. This mechanistic distinction makes CAIA particularly valuable for investigating antibody-mediated inflammation, FcγR signaling, complement-dependent pathways, myeloid-cell activation, and therapeutic targets acting at the downstream effector stage of rheumatoid arthritis-like joint inflammation [14,64].

### 3.3. Human Tumor Necrosis Factor Transgenic Mouse Model

The hTNF-Tg model differs fundamentally from both CIA and CAIA because arthritis develops from chronic overexpression of human TNF rather than immunization or antibody transfer. Persistent TNF signaling creates a sustained inflammatory environment that activates synovial and myeloid compartments and promotes progressive synovitis, pannus formation, cartilage damage, and bone erosion. The original development of the hTNF-Tg model demonstrated that deregulated expression of human TNF-α is sufficient to produce chronic inflammatory polyarthritis and that TNF neutralization can prevent disease development [18]. Importantly, subsequent studies comparing different experimental arthritis models demonstrated that TNF-transgenic arthritis is largely independent of adaptive immune mechanisms, supporting the concept that T- and B-cell responses are not required for disease initiation in this model [68]. Nevertheless, lymphocytes can modulate and amplify inflammation once disease is established. Macrophages represent a major cellular population within hTNF-Tg inflamed joints. Continuous exposure to TNF promotes macrophage activation and production of additional inflammatory mediators, establishing a positive-feedback loop that sustains synovial inflammation. Studies show that TNF blockade rapidly reduces Ly6C^+^ macrophages in hTNF-Tg joints, accompanied by significant clinical and histological improvement. Infliximab treatment also decreases monocyte recruitment into the joint. It also reduces macrophage populations, showing that continuous TNF-dependent monocyte recruitment and macrophage accumulation are key components of established inflammatory disease [69].

Neutrophils are also prominent effector cells in hTNF-Tg arthritis. TNF-driven inflammatory signaling promotes chemokine and adhesion molecule production that facilitates granulocyte recruitment into inflamed joints. Neutrophils can subsequently contribute to tissue injury through proteases, reactive oxygen species, and other inflammatory mediators. The inflammatory cellular network in arthritic synovium includes granulocytes together with macrophages, monocytes, dendritic cells, and lymphocytes, consistent with the development of a multicellular inflammatory response following persistent TNF signaling [1,68].

Monocytes also contribute to hTNF-Tg arthritis. TNF-dependent inflammatory signaling promotes recruitment of circulating monocytes into the inflamed joint, where they contribute to expansion of the macrophage compartment. Experimental TNF blockade markedly decreases monocyte influx into the ankle joints and reduces CCL2 production, providing direct evidence that the monocyte–macrophage axis contributes to maintenance of TNF-driven synovial inflammation [69]. These findings support a model in which persistent TNF signaling continuously promotes myeloid-cell recruitment and differentiation, thereby sustaining inflammatory amplification within the joint. Osteoclasts represent a critical downstream population responsible for the characteristic bone erosions of hTNF-Tg arthritis. TNF promotes osteoclast differentiation and activation both directly and indirectly through inflammatory stromal-cell pathways. Redlich et al. demonstrated the importance of osteoclasts in TNF-mediated joint destruction by showing that hTNF-Tg mice lacking osteoclasts developed severe inflammatory arthritis but were protected from bone destruction [36]. These findings demonstrate that osteoclasts are essential downstream effectors of TNF-mediated skeletal destruction rather than primary initiators of synovial inflammation. The coupling of chronic cytokine-driven synovitis to osteoclast activation provides an important mechanistic basis for the progressive bone erosion observed in the hTNF-Tg model [36].

Thus, hTNF-Tg arthritis can be characterized as a cytokine-driven, predominantly myeloid-mediated model in which persistent TNF signaling activates macrophages, monocytes, neutrophils, synovial/stromal cells, and osteoclastogenic pathways. Although adaptive lymphocytes are not required for the initiation of disease, they can modulate the inflammatory response after disease establishment. The model is therefore particularly useful for investigating chronic TNF-dependent inflammation, myeloid-cell recruitment and activation, inflammatory bone destruction, and therapeutic strategies targeting TNF-associated inflammatory pathways [18,36,68,69].

### 3.4. Zymosan-Induced Arthritis in the SKG Mouse Model

The SKG model provides a distinct representation of autoimmune arthritis because disease originates from a genetically determined defect in T-cell receptor (TCR) signaling and thymic selection. The point mutation in ZAP-70 reduces TCR signaling strength and alters both positive and negative thymic selection, allowing autoreactive CD4^+^ T cells to escape central tolerance. These autoreactive T cells subsequently become pathogenic under appropriate environmental or innate immune stimulation, including exposure to microbial β-glucans such as mannan or curdlan. Unlike CAIA, where adaptive antigen-specific immunity is largely bypassed, and hTNF-Tg arthritis, where TNF-driven inflammation can develop independently of adaptive immune initiation, SKG arthritis is fundamentally T-cell-dependent. Adoptive-transfer studies further demonstrate that SKG CD4^+^ T cells can transfer arthritogenic activity to lymphopenic recipients, supporting the central role of autoreactive T cells in disease induction [19,24,70].

Among CD4^+^ T-cell subsets, Th17 cells are particularly important in SKG arthritis. Autoreactive Th17 cells produce IL-17A and other inflammatory mediators that promote recruitment and activation of neutrophils, macrophages, and other myeloid cells within the joint. SKG studies have demonstrated that self-reactive CD4^+^ T cells preferentially differentiate into IL-17-producing Th17 cells and that IL-17 contributes substantially to chronic autoimmune joint inflammation. Environmental stimulation promotes this process through innate immune activation and cytokine production, including IL-6-dependent pathways that support Th17 differentiation. Furthermore, IL-17A-deficient SKG mice exhibit markedly reduced arthritis, supporting a pathogenic role for IL-17A in disease development [44,54,70]. Thus, the Th17 compartment functions as a critical bridge between autoreactive adaptive immunity and downstream innate inflammation. Pathogenic CD4^+^ T cells can also acquire a GM-CSF-producing phenotype, which further promotes activation of inflammatory myeloid cells. GM-CSF enhances the inflammatory activity of macrophages and monocytes and contributes to the production of mediators such as IL-1β and IL-6, thereby establishing a positive-feedback loop between pathogenic T cells and innate immune cells. In SKG arthritis, GM-CSF-dependent interactions among autoreactive Th17 cells, synovial stromal cells, innate lymphoid cells, and myeloid populations contribute to persistent and amplified joint inflammation. Importantly, experimental studies indicate that although pathogenic CD4^+^ T cells can produce GM-CSF, GM-CSF from non-T-cell populations can also be critical for arthritis development, emphasizing the interconnected nature of the T-cell–myeloid cytokine network [70,71].

Regulatory T cells (Tregs) are also important in SKG arthritis because defective regulation of autoreactive T cells contributes to loss of peripheral tolerance. The ZAP-70 mutation affects not only the selection of autoreactive conventional T cells but also the development and function of Foxp3^+^ regulatory T cells. Consequently, the balance between pathogenic effector T cells and regulatory T cells can become insufficient to restrain autoreactive Th17 responses. Experimental studies show that manipulating the Th17/Treg balance can influence SKG arthritis, with increased Foxp3^+^ Treg responses and reduced IL-17-producing T cells associated with protection from disease [24,70,72].

B cells and plasma cells are also present in SKG arthritis and may contribute to the chronic phase by producing autoantibodies. However, unlike CIA, SKG arthritis does not require antibody-mediated initiation. Pathogenic CD4^+^ T cells can transfer the disease, demonstrating that autoreactive T cells are sufficient to initiate arthritis under appropriate recipient and environmental conditions. SKG mice can nevertheless develop rheumatoid factor and other antibody responses during disease, indicating that B-cell activation may contribute to chronic immune activation and disease amplification even though antibodies are not the primary initiating mechanism [19,24,70].

After autoreactive T-cell activation, macrophages, monocytes, neutrophils, and other innate cells are recruited into the inflamed joint. Neutrophils contribute to tissue injury and inflammatory amplification. In contrast, macrophages respond to T-cell- and stromal-cell-derived cytokines, including IL-17- and GM-CSF-associated inflammatory signals, and produce additional mediators such as IL-1β, IL-6, and TNF. Dendritic cells contribute to antigen presentation and regulate local T-cell responses, while synovial stromal cells produce cytokines and chemokines and help maintain inflammatory cell recruitment. Osteoclast activation downstream of the inflammatory cytokine environment contributes to progressive bone erosion. In particular, IL-17 and GM-CSF mediate key communication among autoreactive T cells, synovial stromal cells, innate lymphoid cells, macrophages, and other myeloid populations, thereby converting an adaptive autoimmune response into sustained tissue inflammation and bone-destructive pathology [54,70,71]. Thus, SKG arthritis is initiated by autoreactive T cells but ultimately reflects sustained interactions between adaptive lymphocytes and innate effector populations.

### 3.5. Overall Comparative Perspective

The dominant cellular compartments differ across the four models: CIA integrates T cells, B cells, dendritic cells, macrophages, neutrophils, and osteoclasts; CAIA emphasizes neutrophils and macrophages downstream of antibody–FcγR/complement activation; hTNF-Tg is characterized by persistent activation of myeloid and stromal compartments with prominent osteoclast-mediated bone destruction; and SKG links autoreactive CD4^+^ T cells, Th17 cells, and inflammatory myeloid populations. These differences provide a practical basis for model selection. CIA or SKG can address adaptive and T-cell-dependent mechanisms, CAIA is suited to antibody-mediated effector pathways, and hTNF-Tg is particularly informative for chronic TNF-dependent inflammation and structural damage. Because human RA involves overlapping and heterogeneous mechanisms, results are best interpreted across complementary models rather than in isolation (Figure 2).

## 4. Inflammatory Mediators’ Involvement Across Experimental Models in CIA, CAIA, hTNF, and SKG

Inflammatory mediators are shared across experimental arthritis models, but their position in the pathogenic cascade differs depending on how the disease is initiated and which immune compartments dominate. TNF-α, IL-1β, IL-6, IL-17A, GM-CSF, CXCL1, CXCL2, CCL2, and RANKL recur across models, but their relative importance and hierarchy differ [7,11,73]. CIA integrates antigen-specific adaptive immunity with downstream innate inflammation; CAIA is dominated by antibody–FcγR/complement effector pathways; hTNF-Tg places TNF-α upstream of a broader inflammatory network; and SKG centers on autoreactive T cells and the IL-23/IL-17 axis. These distinctions are important when interpreting cytokine-targeted interventions because activity in one model may reflect the pathway used to initiate that model rather than a universal feature of RA biology (Table 3).

### 4.1. Mouse Collagen-Induced Arthritis

The CIA model reproduces several important features of RA, including loss of immune tolerance, production of anti-CII antibodies, synovial inflammation, cartilage destruction, and bone erosion. Following CII immunization, antigen-specific T and B cells become activated, leading to the production of pathogenic anti-collagen antibodies and the development of inflammatory responses within the joints. This adaptive immune response is reinforced by innate inflammatory mechanisms, resulting in a complex cytokine network involving TNF-α, IL-1β, IL-6, IL-17A, IL-23, GM-CSF, and RANKL [8,56,74].

TNF-α is a major inflammatory mediator in CIA and contributes to endothelial activation, leukocyte recruitment, synovial inflammation, and tissue destruction. Studies examining cytokine expression in CIA joints demonstrate that TNF-α, IL-1β, and IL-6 are prominently expressed in the synovial lining and at sites adjacent to cartilage and bone, particularly within pannus and erosive lesions. TNF-α also promotes the production of additional inflammatory mediators, including IL-1β, IL-6, and chemokines, thereby establishing a positive-feedback inflammatory circuit [75]. IL-1β contributes particularly to cartilage degradation and bone destruction by stimulating matrix-degrading enzymes and promoting osteoclast-mediated tissue damage. Indeed, experimental blockade of IL-1 in CIA markedly reduces cartilage and bone destruction, highlighting IL-1’s important contribution to structural joint damage [76,77]. The combined actions of TNF-α and IL-1β therefore provide an important mechanistic link between inflammatory signaling and structural joint damage.

IL-6 has an important role in sustaining inflammation and shaping adaptive immunity in CIA. In addition to its direct proinflammatory effects, IL-6 promotes differentiation of naïve CD4+ T cells toward the Th17 lineage in an appropriate cytokine environment. In CIA, IL-6 receptor blockade suppresses Th17 induction and reduces arthritis development, providing direct evidence for the importance of the IL-6–Th17 axis during disease initiation [78]. IL-17A subsequently acts on synovial and stromal cells to enhance inflammatory cytokine and chemokine production, while also promoting granulopoiesis, neutrophil recruitment, and bone destruction. IL-17A can act synergistically with TNF-α to induce IL-6 and RANKL, thereby amplifying both inflammatory and osteoclastogenic pathways [79]. IL-23 further contributes to the maintenance and pathogenic activity of Th17 cells in experimental autoimmune arthritis. In CIA, IL-23 promotes Th17-associated responses and enhances IL-17A production, thereby sustaining IL-17-dependent inflammation [80].

GM-CSF represents another important component of the CIA cytokine network. Produced by activated T cells and other immune populations, GM-CSF promotes inflammatory myeloid-cell responses and contributes to the pathogenic activity of monocytes/macrophages. Experimental evidence shows that GM-CSF administration exacerbates CIA. In contrast, GM-CSF-deficient mice are strongly protected from disease, supporting a critical role for this cytokine in inflammatory arthritis [81,82]. Furthermore, therapeutic neutralization of GM-CSF after disease onset reduces arthritis severity, cartilage destruction, and local TNF-α and IL-1β production, suggesting that GM-CSF contributes to the effector phase of CIA [82]. These findings support a model in which GM-CSF-dependent myeloid activation reinforces the production of downstream inflammatory mediators and sustains synovial inflammation. RANKL provides a critical connection between inflammation and bone destruction by promoting osteoclast differentiation and activation. In inflammatory arthritis, activated T cells, synovial fibroblasts, and other stromal populations can express RANKL, while cytokines such as TNF-α, IL-1β, IL-6, and IL-17 can enhance osteoclastogenic pathways. In CIA, blocking RANKL signaling protects against inflammatory bone erosion, highlighting the importance of the RANKL–RANK axis in osteoclast-mediated bone destruction [36,83]. Moreover, IL-17A and TNF-α can synergistically induce RANKL expression, further linking inflammatory cytokine signaling to osteoclast activation and bone loss [84].

Thus, CIA can be viewed as a multicellular and multidirectional cytokine network in which collagen-specific adaptive immunity initiates disease, while innate immune activation and inflammatory mediators cooperate to sustain synovitis and structural damage. TNF-α, IL-1β, IL-6, IL-17A, GM-CSF, IL-23, and RANKL participate in interconnected pathways involving T cells, B cells, macrophages, neutrophils, dendritic cells, synovial fibroblasts, and osteoclasts. This complexity makes CIA particularly useful for studying the interactions between adaptive and innate immune mechanisms and for evaluating therapies directed against multiple inflammatory pathways [6].

### 4.2. Mouse Collagen Antibody-Induced Arthritis

CAIA provides a model in which antibody-mediated mechanisms and innate immune activation are prominent, while the requirement for antigen-specific T-cell priming is substantially reduced [13,51]. The resulting cytokine network differs from that observed in CIA and is characterized by strong contributions from TNF-α, IL-1β, IL-6, chemokines, and complement-associated inflammatory pathways [27,85]. After pathogenic immune complexes deposit in the joint, Fc receptor-dependent activation of innate immune cells, together with complement activation, promotes the recruitment and activation of neutrophils, monocytes, and macrophages [27,65]. These cells produce TNF-α, IL-1β, IL-6, and other inflammatory mediators. TNF-α contributes to endothelial activation, leukocyte recruitment, and amplification of synovial inflammation, while IL-1β promotes tissue destruction and osteoclast activation [86]. IL-6 contributes to the inflammatory environment and can further stimulate stromal and myeloid-cell responses [53].

Chemokines are particularly important in CAIA because rapid recruitment of innate immune cells is a major feature of disease development. CXCL1 and CXCL2 contribute to neutrophil recruitment, whereas CCL2 promotes recruitment of monocytes and macrophages [27,87]. The accumulation of these inflammatory cells establishes a feed-forward loop in which infiltrating myeloid cells produce additional TNF-α, IL-1β, IL-6, and other inflammatory mediators. GM-CSF can further promote inflammatory myeloid-cell activation and has been implicated in maintaining inflammatory arthritis. However, its contribution is better established in other experimental arthritis models than as a primary initiating mediator of CAIA [88,89].

The IL-23/IL-17 axis is generally less dominant in CAIA than in CIA because the model does not require the development of a collagen-specific adaptive T-cell response for disease induction. This distinction matters when interpreting studies of IL-17- or IL-23-directed therapies. CIA involves substantial contributions from collagen-specific Th17 responses, and IL-17A has been shown to promote joint inflammation, cartilage destruction, and bone erosion in this model [90,91]. In contrast, CAIA bypasses the antigen-specific T-cell priming phase and primarily models the downstream antibody-mediated effector phase of inflammatory arthritis [27]. Therefore, a limited response to IL-17- or IL-23-directed intervention in CAIA does not necessarily mean these pathways are unimportant in rheumatoid arthritis; rather, it may reflect the antibody-driven mechanism that initiates the disease. RANKL-mediated osteoclastogenesis remains relevant to the development of bone-erosive pathology. In inflammatory arthritis, activated immune and stromal cells can increase RANKL expression, promoting osteoclast differentiation and bone resorption [83,92]. Thus, CAIA can be used to investigate mechanisms linking antibody-mediated inflammation and innate immune activation to downstream cartilage and bone destruction. Overall, CAIA represents an antibody–Fc receptor/complement–innate cytokine axis, with TNF-α, IL-1β, IL-6, CXCL1, CXCL2, and CCL2 contributing to the recruitment and activation of inflammatory myeloid cells [27,65,85]. Its relative independence from antigen-specific T-cell priming makes CAIA particularly useful for investigating antibody-mediated inflammation, Fc receptor signaling, complement activation, neutrophil recruitment, and downstream innate effector mechanisms. This mechanistic distinction also makes CAIA complementary to CIA, with the two models providing opportunities to distinguish pathways involved in adaptive immune initiation from those responsible for antibody-mediated inflammatory effector responses.

### 4.3. Human Tumor Necrosis Factor Transgenic Mouse Model

The hTNF-Tg model provides a fundamentally different cytokine architecture because arthritis is driven by constitutive overexpression of human TNF-α. In contrast to CIA and CAIA, where cytokine production occurs downstream of experimentally induced immune activation, TNF-α itself functions as the primary initiating and sustaining inflammatory stimulus. Persistent TNF signaling activates TNF receptors on immune, stromal, endothelial, and synovial cells and establishes a chronic inflammatory environment that resembles important aspects of TNF-dependent human RA pathology. The original Tg197 model demonstrated TNF-α’s central role: constitutive expression of human TNF-α caused spontaneous chronic inflammatory polyarthritis, while a TNF-neutralizing antibody prevented disease development [18]. Subsequent studies further showed that anti-TNF treatment suppresses disease severity and inflammatory mediator expression in Tg197 mice, supporting TNF-α as the dominant upstream driver of pathology [18].

TNF-α induces the production of multiple secondary inflammatory mediators, including IL-1β and IL-6, and promotes the expression of chemokines involved in inflammatory leukocyte recruitment [18,93,94]. CXCL1 and CXCL2 contribute to recruitment of neutrophils and other myeloid cells, whereas CCL2 facilitates monocyte/macrophage recruitment. These infiltrating cells subsequently produce additional inflammatory cytokines and tissue-destructive mediators, creating a feed-forward inflammatory circuit downstream of TNF signaling. Consistent with this organization, inflammatory joints from Tg197 mice show increased IL-1β and IL-6 expression, demonstrating that TNF-driven inflammation is associated with amplification of the local cytokine network [94].

IL-1β contributes substantially to cartilage and bone pathology and promotes inflammatory activation of synovial and myeloid cells. In contrast, IL-6 appears to contribute more context-dependently in TNF-driven arthritis than in antigen-induced models such as CIA. Although IL-6 increases in inflamed Tg197 joints, genetic studies show that IL-6 is not essential for the development of severe TNF-driven arthritis, emphasizing that IL-6 is a downstream component rather than the primary initiating cytokine in this model [95]. GM-CSF can additionally contribute to activation and expansion of inflammatory myeloid populations and therefore provides another potential link between TNF-driven inflammation and downstream cellular effector mechanisms [88].

TNF-α additionally promotes osteoclast-mediated bone destruction through mechanisms involving RANKL and other osteoclast-supporting pathways. In the hTNF-Tg model, inflammatory arthritis is accompanied by extensive osteoclast formation and progressive bone erosion. Importantly, genetic elimination of c-Fos, which prevents functional osteoclast development, protects hTNF-Tg mice from TNF-mediated bone erosion despite persistent inflammatory arthritis, demonstrating that osteoclasts are essential effectors of TNF-induced joint destruction [40]. These findings establish an important mechanistic connection between persistent TNF signaling, osteoclastogenesis, and progressive bone erosion.

Thus, the hTNF-Tg model illustrates how sustained activity of a single dominant cytokine can generate a broader inflammatory network involving IL-1β, IL-6, chemokines, inflammatory myeloid populations, and osteoclastogenic pathways [18,36,94]. In contrast to CIA, where antigen-specific adaptive immune responses are central to disease initiation, the hTNF-Tg model is primarily driven by constitutive cytokine signaling and does not require antigen-specific immunization for arthritis development. IL-17A, IL-12, and IFN-γ therefore do not occupy the same upstream position as TNF-α in initiating disease in this model. Consequently, the hTNF-Tg model is particularly valuable for investigating TNF-dependent chronic inflammation, synovial pathology, inflammatory bone destruction, and therapeutic approaches targeting TNF signaling [18]. However, consider its cytokine architecture when interpreting therapies directed toward pathways more dependent on antigen-specific adaptive immunity, because efficacy in a TNF-driven model may not predict efficacy in models that require T-cell and B-cell responses for disease initiation.

### 4.4. Zymosan-Induced Arthritis in the SKG Mouse Model

SKG arthritis is strongly associated with Th17-driven inflammation and therefore has a cytokine network that differs substantially from the TNF-dominant hTNF-Tg model. IL-17A is a central effector cytokine in SKG arthritis. Pathogenic Th17 cells produce IL-17A, which acts on synovial fibroblasts, macrophages, endothelial cells, and other stromal cells to induce inflammatory mediators and chemokines, promote neutrophil and monocyte recruitment, and amplify local tissue inflammation. IL-23 supports the maintenance and pathogenic function of Th17 cells, establishing an IL-23/IL-17 axis that is central to disease progression [96].

IL-6 and TGF-β contribute to the differentiation and polarization of naïve CD4+ T cells toward the Th17 lineage, placing IL-6 and TGF-β upstream of IL-17A in the development of pathogenic T-cell responses, whereas IL-23 is particularly important for sustaining pathogenic Th17 activity. This hierarchical organization distinguishes SKG arthritis from the hTNF-Tg model, in which TNF-α is the dominant initiating cytokine [44].

GM-CSF produced by pathogenic T cells also contributes to inflammatory myeloid-cell activation. GM-CSF-responsive myeloid cells can subsequently produce TNF-α, IL-1β, IL-6, and other inflammatory mediators, establishing a reciprocal interaction between pathogenic T cells and innate immune cells. TNF-α and IL-1β therefore remain important downstream amplifiers of inflammation even though they are not the principal initiating drivers of the SKG phenotype [97].

The regulatory cytokine IL-10 provides an important counterbalance to these inflammatory pathways and can suppress excessive immune activation. The balance between inflammatory cytokines and regulatory mechanisms may therefore influence disease severity and chronicity in SKG arthritis. Overall, SKG arthritis can be characterized as a T-cell/Th17–myeloid cytokine network in which altered T-cell signaling promotes pathogenic Th17 responses, followed by IL-17A-, GM-CSF-, TNF-α-, and IL-1β-dependent activation of inflammatory myeloid and stromal compartments [98,99].

### 4.5. Comparative Analysis of Inflammatory Mediators

Although CIA, CAIA, hTNF-Tg, and SKG arthritis share several inflammatory mediators, the biological hierarchy of these cytokines differs considerably among the models. CIA has one of the most complex cytokine profiles because the disease involves both collagen-specific adaptive immunity and secondary innate inflammatory responses. TNF-α, IL-1β, IL-6, IL-17A, IL-23, GM-CSF, and RANKL participate at different stages of disease development. The IL-6/IL-23/IL-17 axis is particularly important for linking adaptive T-cell responses to downstream tissue inflammation. In contrast, TNF-α and IL-1β provide major inflammatory and tissue-destructive functions. CAIA shifts this balance toward innate immunity. Because pathogenic antibodies initiate disease, Fc receptor- and complement-mediated activation of neutrophils and macrophages occurs without requiring a primary collagen-specific T-cell response. Consequently, TNF-α and IL-1β are prominent downstream cytokines, while CXCL1, CXCL2, and CCL2 are particularly important for recruiting inflammatory myeloid cells. GM-CSF further supports myeloid activation. Compared with CIA, the IL-23/IL-17 axis is less central to disease initiation and progression. The hTNF-Tg model represents the clearest single-cytokine-dominant system among the four models. TNF-α is constitutively elevated and drives the production of secondary inflammatory mediators, including IL-1β, IL-6, GM-CSF, CXCL1, CXCL2, and CCL2. This hierarchical relationship matters experimentally because inhibiting TNF-α can suppress multiple downstream inflammatory pathways simultaneously. Conversely, targeting an individual downstream cytokine may not completely suppress disease because the initiating TNF signal remains active.

SKG arthritis is more strongly organized around the Th17/IL-17 cytokine axis. IL-6 and TGF-β contribute to Th17 differentiation, IL-23 supports pathogenic Th17 responses, and IL-17A drives downstream activation of stromal and myeloid cells. GM-CSF, TNF-α, and IL-1β subsequently contribute to inflammatory amplification. Thus, SKG arthritis is particularly informative for investigating the interaction between pathogenic T cells and inflammatory myeloid responses (Figure 3).

## 5. Therapeutic Mechanisms in CIA, CAIA, hTNF-Tg, and SKG

### 5.1. Mouse Collagen-Induced Arthritis

CIA is an active autoimmune model that generates both CII-specific T-cell and B-cell responses. CD4^+^ T cells provide help to B cells, resulting in the production of pathogenic anti-CII antibodies. These antibodies subsequently activate complement and Fcγ receptors and recruit innate immune cells into the joint, thereby amplifying local inflammation and tissue injury [100,101]. IL-23-dependent Th17 responses are particularly important in CIA, whereas IL-12-driven Th1 responses are less critical and can, under some experimental conditions, be protective [102,103].

Therapeutically, CIA therefore provides an opportunity to evaluate interventions at multiple stages of the autoimmune and inflammatory cascade. Methotrexate (MTX), a conventional disease-modifying antirheumatic drug (csDMARD), suppresses CIA through broad immunomodulatory and anti-inflammatory mechanisms. In murine CIA, MTX treatment reduces disease severity and joint pathology and has been shown to suppress TNF-α production by T cells and macrophages, supporting inhibition of proinflammatory cytokine production as an important component of its therapeutic activity [104]. MTX also interferes with folate-dependent metabolic pathways by inhibiting dihydrofolate reductase and other folate-dependent enzymes, while low-dose anti-inflammatory activity is associated with increased extracellular adenosine signaling and modulation of inflammatory-cell function [105]. More recent CIA studies demonstrate that MTX efficacy is dose-dependent and is associated with alterations in tissue folate metabolism, further supporting the contribution of folate-dependent mechanisms to its activity in experimental arthritis [106]. B-cell depletion or inhibition of antibody production can reduce pathogenic autoantibody generation. In contrast, T-cell-directed approaches can suppress autoreactive T-cell activation and Th17 differentiation. Blocking TNF-α, IL-1β, IL-6, or IL-17A can interrupt downstream inflammatory amplification and joint pathology [100,103]. In addition, inhibition of JAK/STAT signaling can suppress signaling downstream of several cytokine receptors simultaneously, providing a broader strategy for modulating cytokine-driven inflammatory networks [107]. Thus, therapeutic efficacy in CIA can reflect inhibition of either the initiating adaptive immune response or the downstream inflammatory effector phase.

### 5.2. Mouse Collagen Antibody-Induced Arthritis

CAIA differs fundamentally from CIA because animals receive preformed anti-CII monoclonal antibodies, bypassing the immunization and antigen-priming phase required to develop collagen-specific adaptive immunity. Consequently, disease induction does not require initial activation and priming of collagen-specific B and T cells or endogenous generation of anti-CII antibodies. Instead, preformed anti-CII antibodies bind type II collagen within the joint and form antibody–antigen complexes that engage Fcγ receptors and activate complement, promoting recruitment and activation of neutrophils, macrophages, and other innate effector cells. This antibody-dependent effector phase is central to CAIA and distinguishes it mechanistically from CIA [14,27].

This mechanistic organization makes CAIA particularly useful for evaluating therapeutics directed against the effector phase of antibody-mediated arthritis. Experimental studies have shown that Fcγ receptor signaling, complement activation, and neutrophil-mediated inflammation contribute significantly to antibody-induced arthritis, supporting the use of CAIA to investigate interventions that target these downstream pathways. In addition, inhibiting inflammatory mediators such as TNF-α and IL-1β can attenuate the inflammatory cascade associated with antibody-mediated joint inflammation. Because CAIA largely bypasses the adaptive immune priming phase, therapeutic efficacy in this model provides useful evidence that a compound can suppress downstream inflammatory and antibody-effector mechanisms rather than primarily interfering with the initiation of antigen-specific autoimmunity [12,22,27].

### 5.3. Human Tumor Necrosis Factor Transgenic Mouse Model

The hTNF-Tg model is characterized by constitutive overexpression of human TNF-α and the spontaneous development of progressive, erosive polyarthritis that closely recapitulates several features of chronic inflammatory arthritis, including synovial hyperplasia, cartilage destruction, and bone erosion [35]. TNF-α functions as an upstream inflammatory driver, promoting activation of synovial fibroblasts, endothelial cells, macrophages, and other inflammatory cell populations. Sustained TNF signaling also induces downstream inflammatory mediators, including IL-1β and IL-6. It contributes to cartilage and bone destruction by activating matrix-degrading pathways and promoting osteoclastogenesis [37]. The therapeutic mechanism in this model therefore centers on TNF-α neutralization. Treatment of hTNF-Tg mice with anti-TNF-α monoclonal antibodies markedly suppresses clinical arthritis, reduces inflammatory mediator expression, and limits pathological progression. Importantly, anti-TNF treatment has shown efficacy even when administered after established arthritis, providing strong pharmacological validation of TNF-α as a critical therapeutic target and demonstrating the model’s utility for evaluating TNF-pathway inhibitors [108].

Beyond direct TNF inhibition, the hTNF-Tg model can be used to investigate downstream inflammatory pathways, including NF-κB and MAPK signaling, IL-1/IL-6-mediated inflammation, matrix metalloproteinase activity, and osteoclast differentiation. Osteoclasts are particularly important because they represent a critical effector population linking TNF-driven inflammation to structural bone destruction. Studies in hTNF-Tg mice showed that eliminating osteoclasts prevents TNF-dependent bone erosion despite persistent synovial inflammation, establishing osteoclasts as essential mediators of TNF-induced joint destruction [36]. In addition, osteoprotegerin-mediated targeting of osteoclast activity reduces TNF-dependent bone destruction, supporting investigation of the RANKL-RANK-OPG axis as a therapeutic pathway in this model [36].

### 5.4. Zymosan-Induced Arthritis in the SKG Mouse Model

SKG mice provide a distinct model of autoimmune arthritis driven by a mutation in ZAP-70, a critical component of T-cell receptor (TCR) signaling. Altered ZAP-70 signaling changes the threshold for thymic positive and negative selection, allowing highly autoreactive CD4+ T cells to escape into the periphery. Following appropriate microbial or β-glucan stimulation, these autoreactive T cells become activated and contribute to systemic autoimmune arthritis [24,54,109]. The therapeutic biology of SKG arthritis is strongly associated with Th17 differentiation and IL-17A signaling. IL-6 promotes the differentiation and expansion of pathogenic Th17 cells, while IL-23 contributes to the maintenance and pathogenicity of Th17 responses. IL-17A promotes the recruitment and activation of inflammatory cells and stimulates stromal cells to produce additional inflammatory mediators, thereby amplifying joint inflammation. Consistent with this mechanism, disrupting IL-6 or IL-17A signaling can substantially reduce disease, while modulating the Treg/Th17 balance through CD4-directed approaches can influence disease development [44,110,111].

An important advantage of SKG arthritis is that it links genetic susceptibility, defective immune tolerance, environmental triggers, autoreactive CD4+ T cells, and Th17-mediated inflammation. Therefore, it can be particularly useful for evaluating therapies aimed at pathogenic T-cell populations and cytokine networks that are difficult to dissect in models dominated primarily by antibody- or TNF-driven inflammation. The SKG model provides an important complementary platform for investigating the cellular and cytokine interactions linking autoreactive T-cell activation to chronic synovial inflammation and tissue pathology [24,44,110].

### 5.5. Comparative Therapeutic Significance

Interpret therapeutic responses in relation to the biology that drives each model. Efficacy in hTNF-Tg supports activity against TNF-dependent inflammation; efficacy in SKG can provide evidence for activity against T-cell/Th17-associated pathways; and efficacy in CAIA is particularly informative for antibody-mediated innate effector mechanisms. CIA offers a broader test of interventions that influence antigen-specific adaptive immunity together with downstream inflammatory pathways. Using mechanistically distinct models can therefore help determine whether a candidate acts on a specific pathogenic pathway or exerts broader anti-inflammatory activity (Figure 4).

## 6. Clinical Scoring in CIA, CAIA, hTNF, and SKG

Clinical scoring systems in preclinical RA models quantify arthritis severity primarily by joint inflammation, typically through measures such as paw swelling, erythema, and the number of affected joints; however, interpretation varies with the underlying disease mechanism, disease kinetics, and experimental design. Importantly, differences in the relative contributions of innate and adaptive immunity among models such as CIA, CAIA, and TNF-transgenic arthritis can influence the extent and nature of joint inflammation and therefore the meaning of clinical scores. Accordingly, interpret clinical scoring within the context of the specific arthritis model and complement it with histopathological assessment of synovial inflammation, cartilage damage, and bone erosion [112,113] (Figure 5).

In the CIA model, clinical scoring reflects the development of autoimmune arthritis mediated by adaptive immune responses to CII, involving both T- and B-cell responses and the subsequent activation of inflammatory effector pathways. Disease severity is commonly assessed by visual examination of each paw, evaluating erythema, swelling, and the extent of joint involvement. Several semiquantitative scoring systems have been described, most commonly assigning scores of 0–4 per paw, although modified 0–5 scales are also used depending on the experimental protocol. Higher scores indicate progressively greater inflammation, paw swelling, joint involvement, and functional impairment. Quantitative paw-thickness measurements and histopathological assessment of synovial inflammation, cartilage damage, and bone erosion typically complement clinical scoring. Because CIA develops following antigen-specific adaptive immune activation, longitudinal clinical scores provide an important measure of disease onset and progression and can be correlated with inflammatory cell infiltration and subsequent structural joint damage [10,56,114,115] (Figure 6).

In the CAIA model, arthritis is initiated by passive transfer of pathogenic anti-CII antibodies, triggering rapid immune-complex formation and Fc-receptor- and complement-dependent inflammatory responses without requiring the antigen-specific T-cell priming that characterizes CIA. Consequently, clinical scoring in CAIA primarily reflects acute inflammatory changes, including paw swelling, erythema, and the number and extent of affected joints. Scoring systems vary among studies and commonly use 0–3 or 0–4 points per paw, while some protocols use a 0–5 scale for regional joint swelling with additional points for digital joint involvement. The maximum score therefore depends on the specific scoring system and number of paws evaluated. CAIA clinical scores are particularly useful for quantifying the magnitude, onset, and duration of acute joint inflammation and for evaluating the efficacy of anti-inflammatory or immunomodulatory therapies targeting established inflammatory effector mechanisms [22,116,117] (Figure 7).

The hTNF-Tg model represents chronic, TNF-driven inflammatory arthritis characterized by persistent synovitis, progressive cartilage degradation, and bone erosion. Clinical assessment typically includes paw and ankle swelling, erythema, joint stiffness, reduced grip strength or mobility, and, in more advanced disease, joint deformity. Unlike CIA and CAIA, where composite paw inflammation scores commonly quantify disease severity, hTNF-Tg studies often include additional measures of functional impairment and structural joint damage, including grip-strength testing, gait analysis, histopathological assessment of cartilage and bone, and micro-computed tomography (microCT). These additional endpoints are particularly relevant because clinical severity in this chronic model reflects not only ongoing inflammation but also progressive structural damage to cartilage and bone. Indeed, functional impairment in hTNF-Tg mice has been shown to correlate more strongly with cartilage damage and bone erosion than with the extent of synovial inflammation, highlighting the importance of incorporating structural and functional endpoints when evaluating disease progression and therapeutic efficacy [118,119] (Figure 8).

The SKG arthritis model, originally developed by Sakaguchi and colleagues, is driven by a point mutation in the ZAP-70 gene that alters T-cell receptor signaling, thymic selection, and peripheral T-cell activation. This genetic alteration results in an autoimmune arthritis model characterized by strong T-cell involvement and either spontaneous or trigger-dependent disease onset [24]. Clinical scoring typically assesses peripheral joint inflammation by measuring paw swelling, redness, and the number of affected joints. Depending on the study protocol, researchers may score disease severity on a 0–5 scale per paw or use joint-based scoring systems [56,118]. Because SKG arthritis involves immune dysregulation and chronic inflammatory progression, researchers often interpret clinical scores alongside histopathological analysis, immune profiling, and measurements of cartilage and bone erosion [24]. Overall, CIA and SKG models are particularly useful for investigating adaptive immune mechanisms. However, CIA is initiated by an antigen-specific response to collagen, whereas SKG arthritis results from genetically altered T-cell signaling. Across models, clinical scores are most informative when paired with objective measures such as paw thickness, histopathology, imaging, immune-cell profiling, or molecular biomarkers. The same numerical score should not be assumed to represent equivalent biological severity across CIA, CAIA, hTNF-Tg, and SKG arthritis because disease kinetics, dominant cell populations, and mechanisms of tissue injury differ among models [1,18,116].

## 7. Conclusions

Preclinical mouse models remain essential for dissecting RA pathogenesis and evaluating therapeutic strategies, but no single model reproduces the full complexity or heterogeneity of human disease. CIA, CAIA, hTNF-Tg, and SKG arthritis represent distinct biological systems that should be selected according to the mechanism under investigation rather than treated as interchangeable models. CIA is particularly informative for antigen-specific adaptive immunity and the interaction of T cells, B cells, autoantibodies, and innate inflammatory pathways. CAIA isolates the antibody-mediated effector phase and highlights FcγR-, complement-, neutrophil-, and macrophage-dependent inflammation. hTNF-Tg captures chronic TNF-driven synovitis and structural damage.

In contrast, SKG links altered T-cell tolerance with environmental stimulation and Th17-associated inflammation. These differences directly affect how we should interpret therapeutic responses. A mechanism-based panel of complementary models, supported by human translational evidence, can provide stronger evidence than reliance on a single experimental system.

Future improvements in RA modeling, including genetically refined and humanized models, patient-derived systems, single-cell and spatial profiling, and multi-omics, may further improve the biological resolution and translational value of preclinical studies.

## Figures and Tables

**Figure 1:**
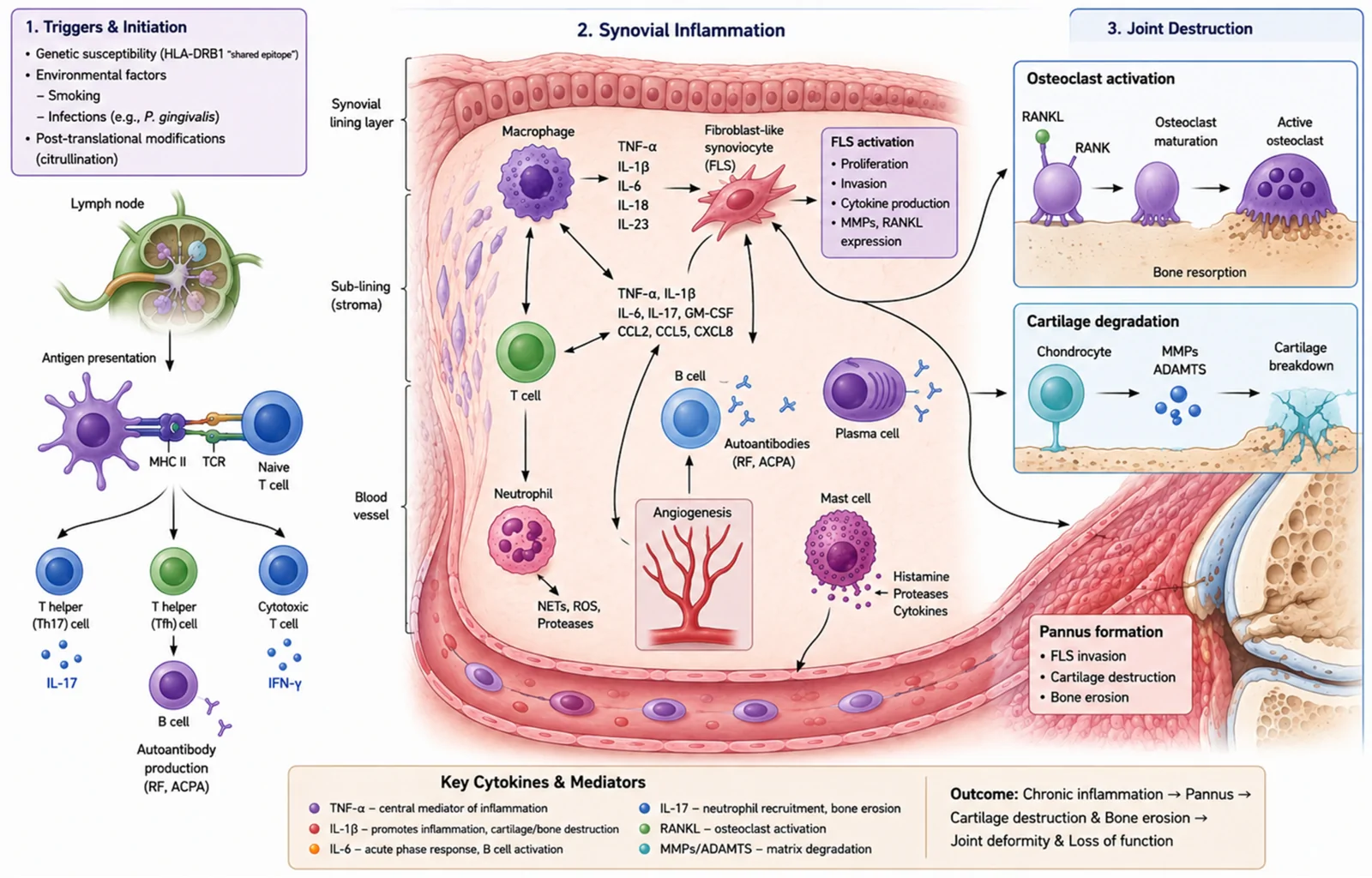
Cellular-Level Overview of Rheumatoid Arthritis: Immune Activation, Inflammatory Crosstalk, and Joint Pathology.

**Figure 2:**
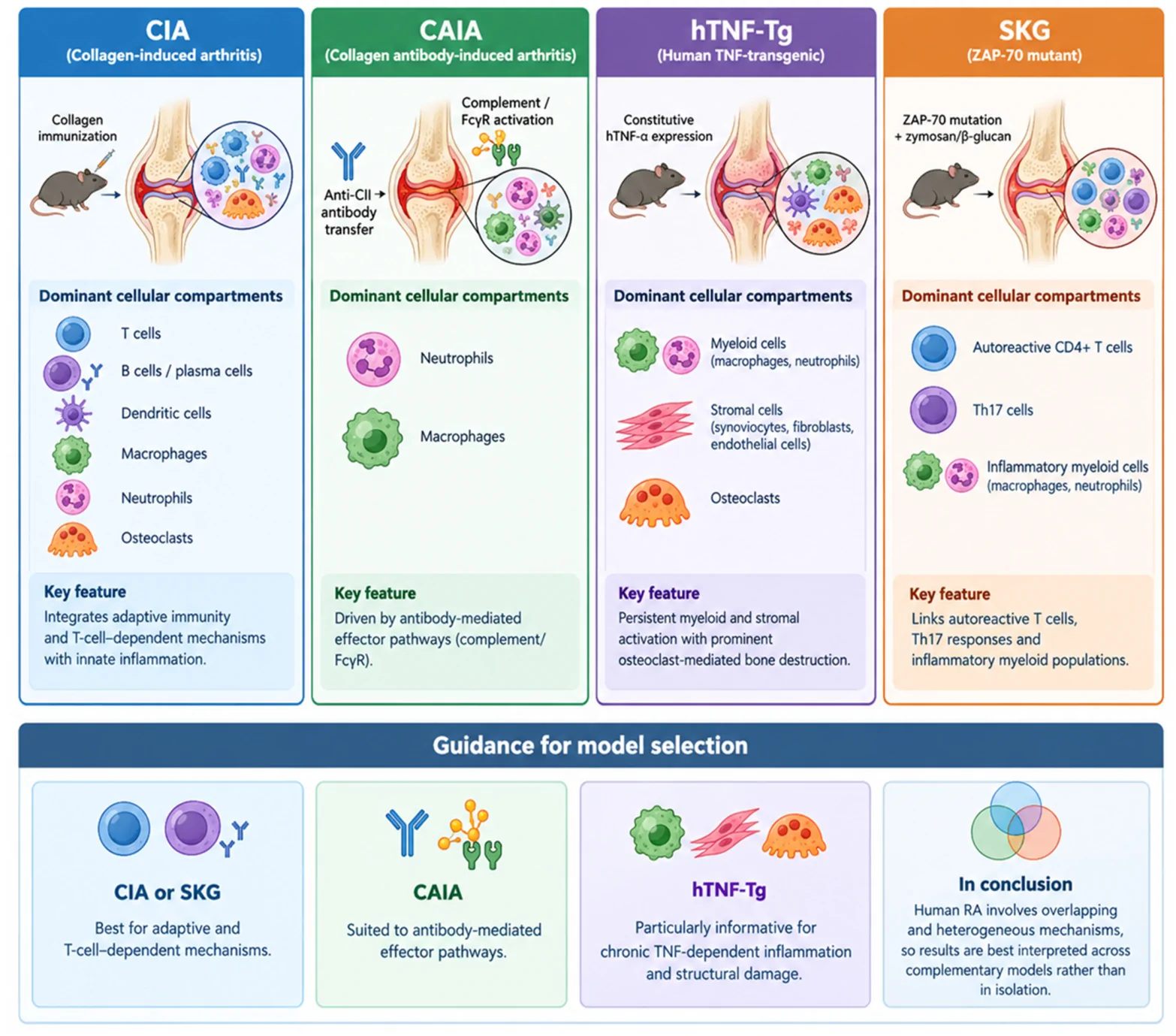
Comparative Immune Cells Across CIA, CAIA, hTNF-Tg, and SKG Arthritis Models.

**Figure 3:**
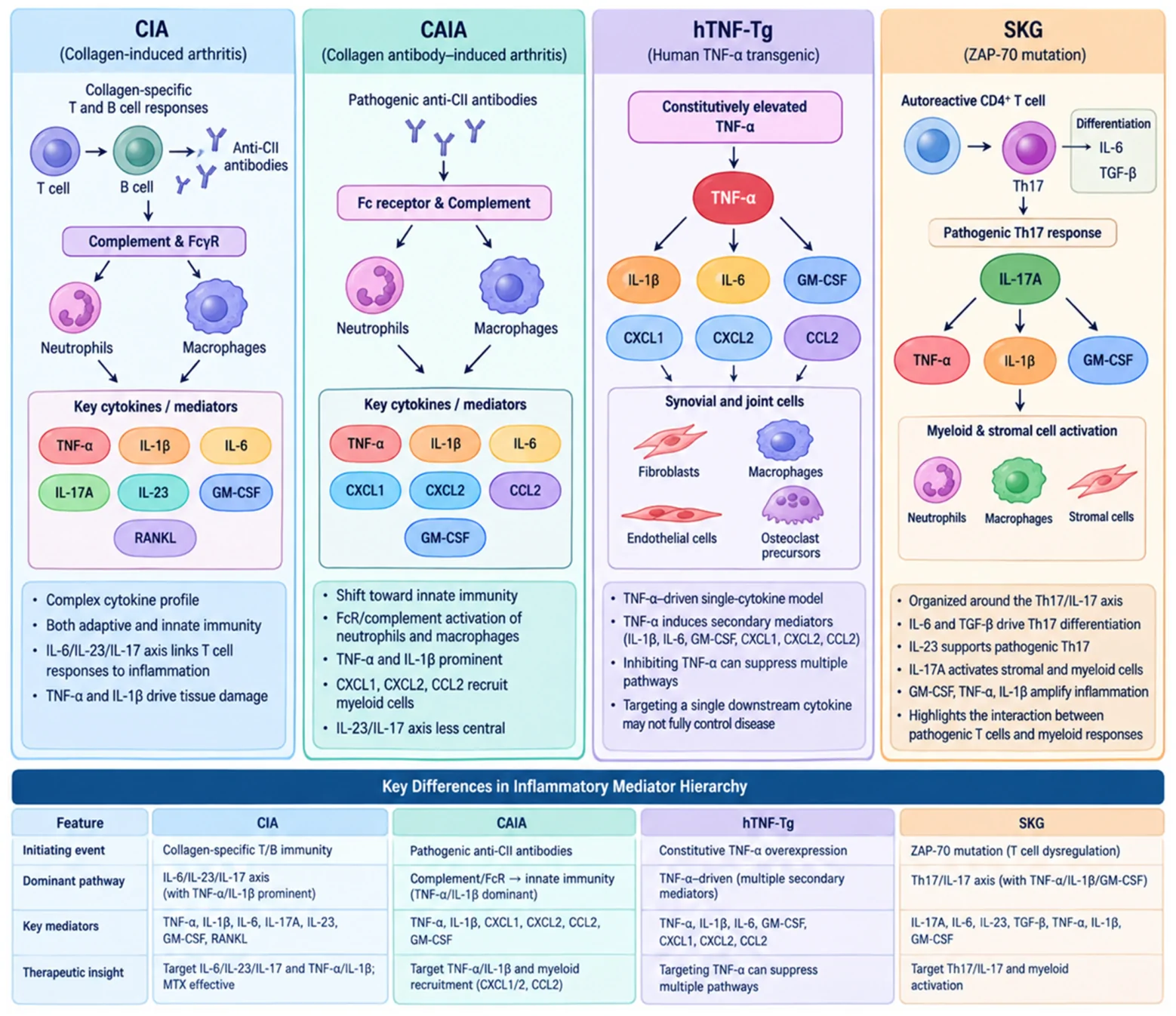
Inflammatory Mediator Hierarchy Across CIA, CAIA, hTNF-Tg, and SKG Arthritis Models.

**Figure 4:**
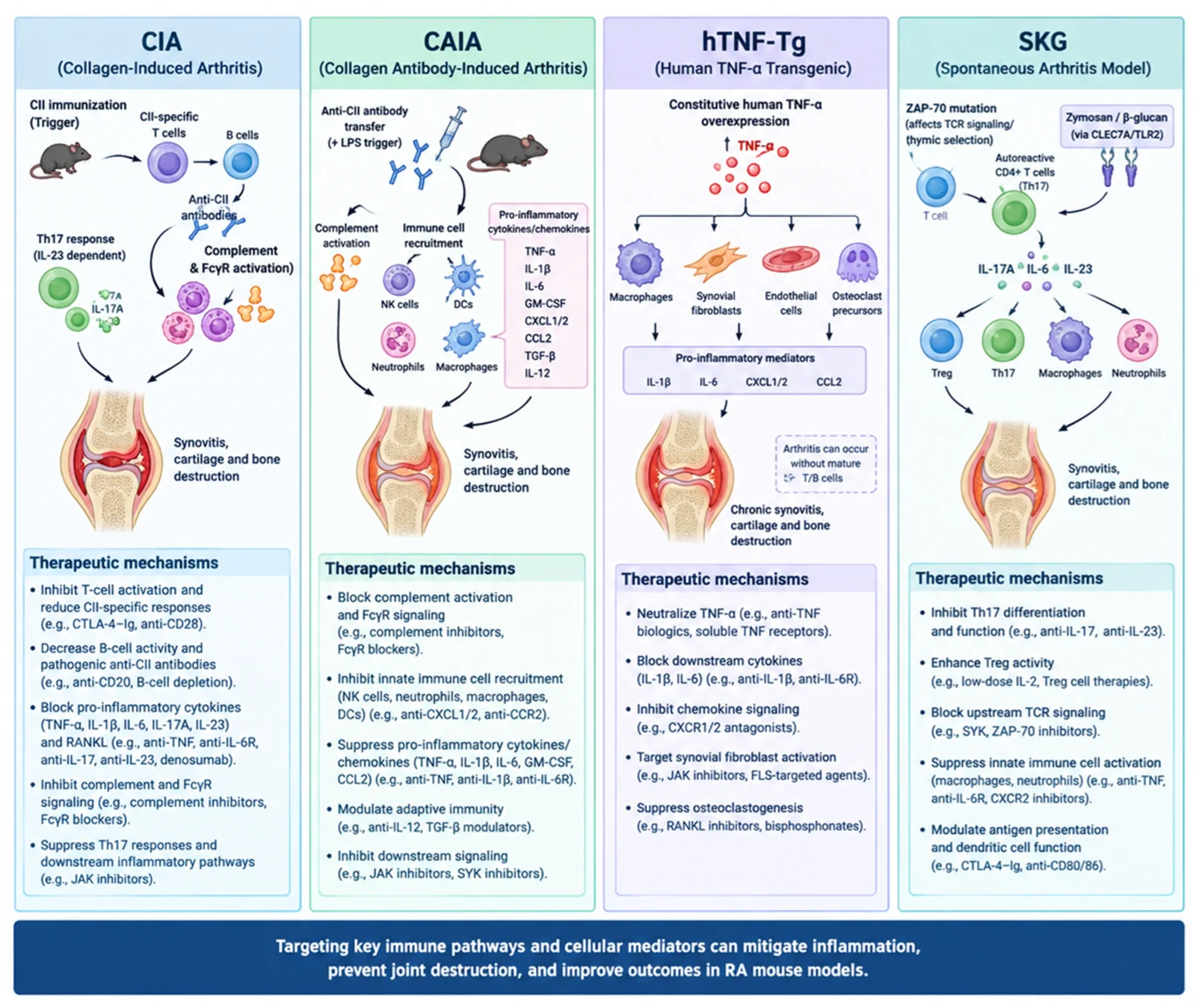
Therapeutic Mechanisms and Targeted Pathways Across CIA, CAIA, hTNF-Tg, and SKG Mouse Models.

**Figure 5:**
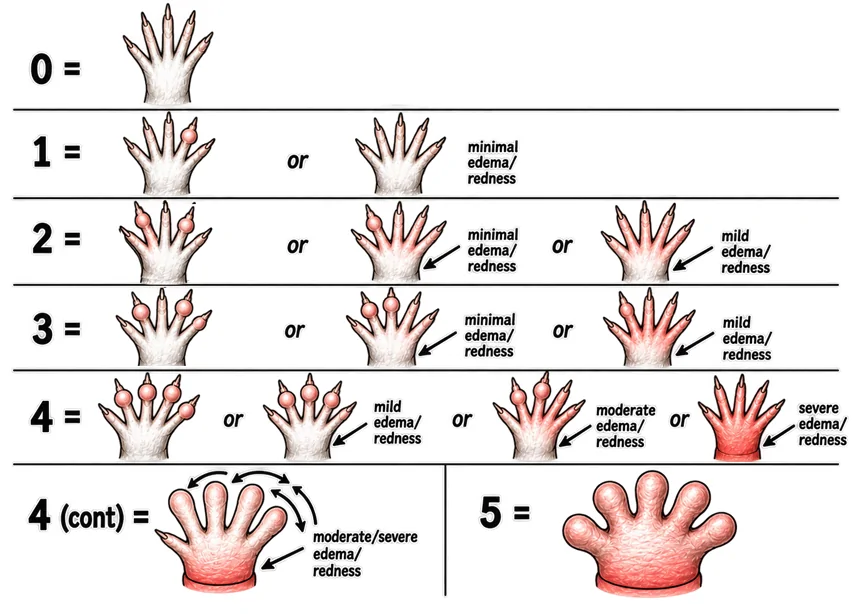
Schematic representation of mouse paw arthritis clinical scoring. Illustrations show progressive changes from score 0 (normal) to score 5 (severe arthritis), characterized by increasing joint swelling, edema, erythema, and extensive paw involvement.

**Figure 6:**
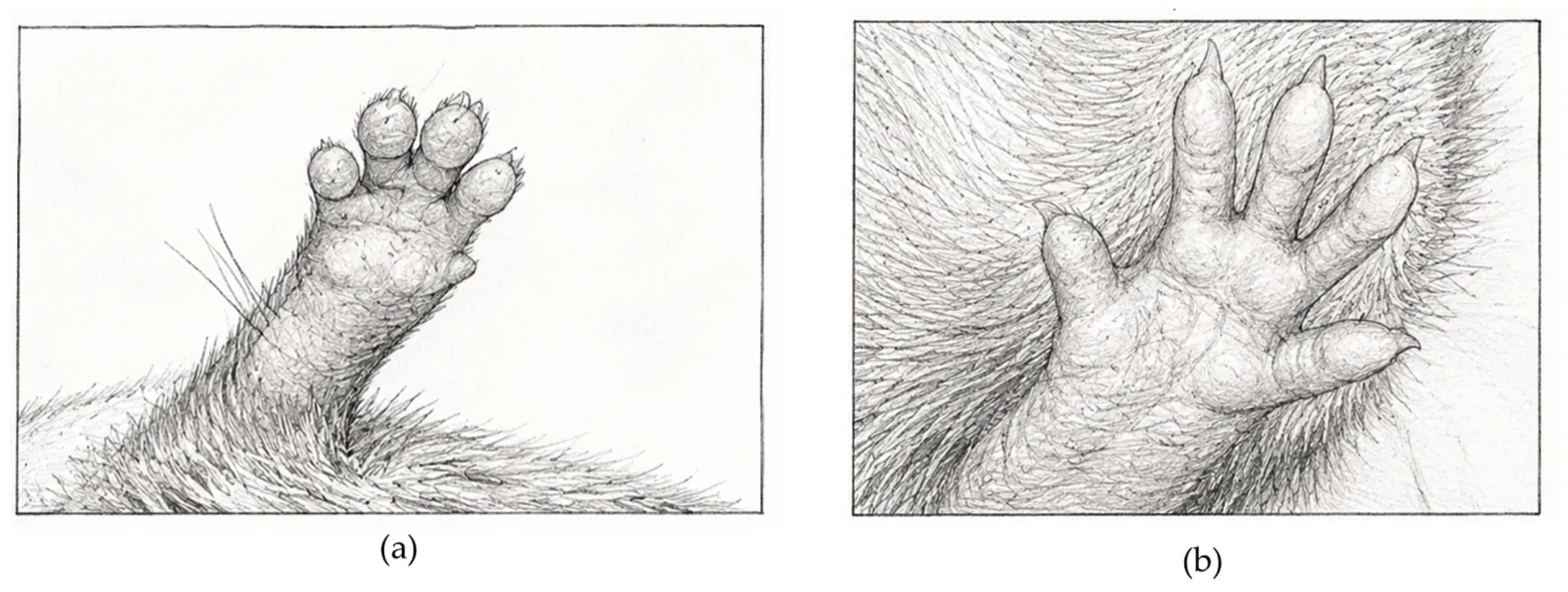
Representative Sketches of (**a**) Forepaw and (**b**) Hindpaw Swelling in the Collagen-Induced Arthritis (CIA) Mouse or Zymosan-induced arthritis in the SKG mouse model. The animals show a swollen digit, associated with swelling of the wrists and ankles.

**Figure 7:**
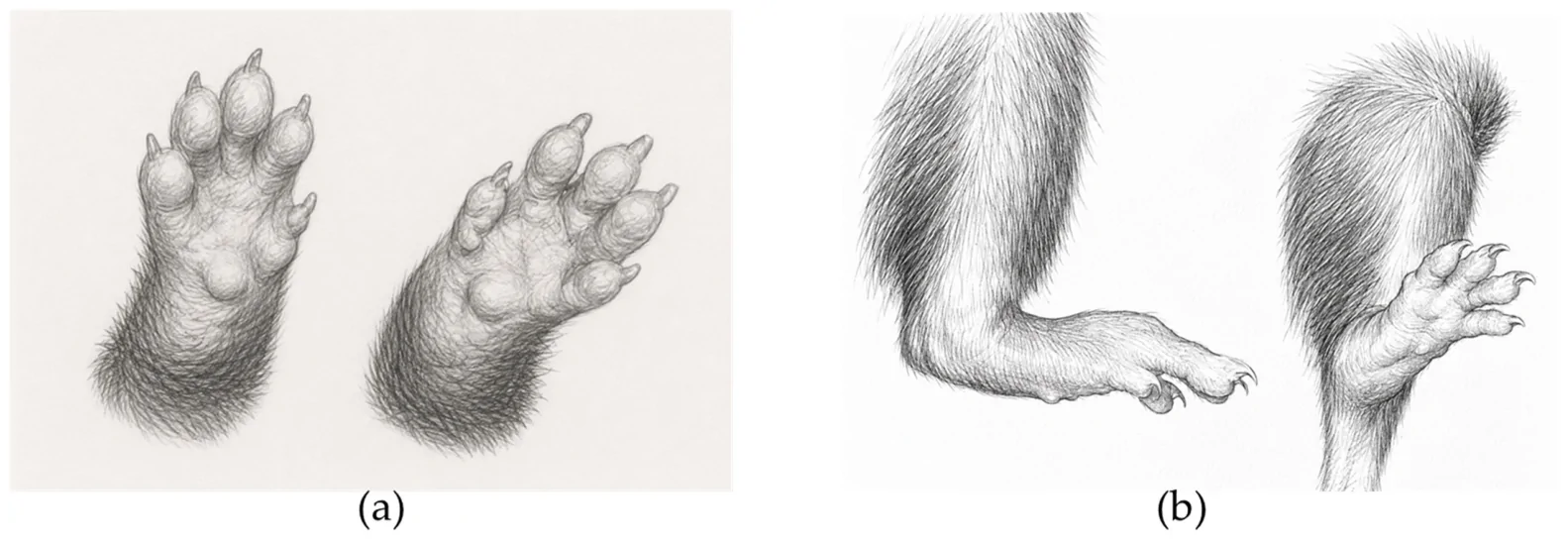
Representative Sketches of (**a**) Forepaw and (**b**) Hindpaw Swelling in the Collagen antibody-Induced Arthritis (CAIA) Mouse. The animals show swollen digits, with swelling of the wrists and ankles.

**Figure 8:**
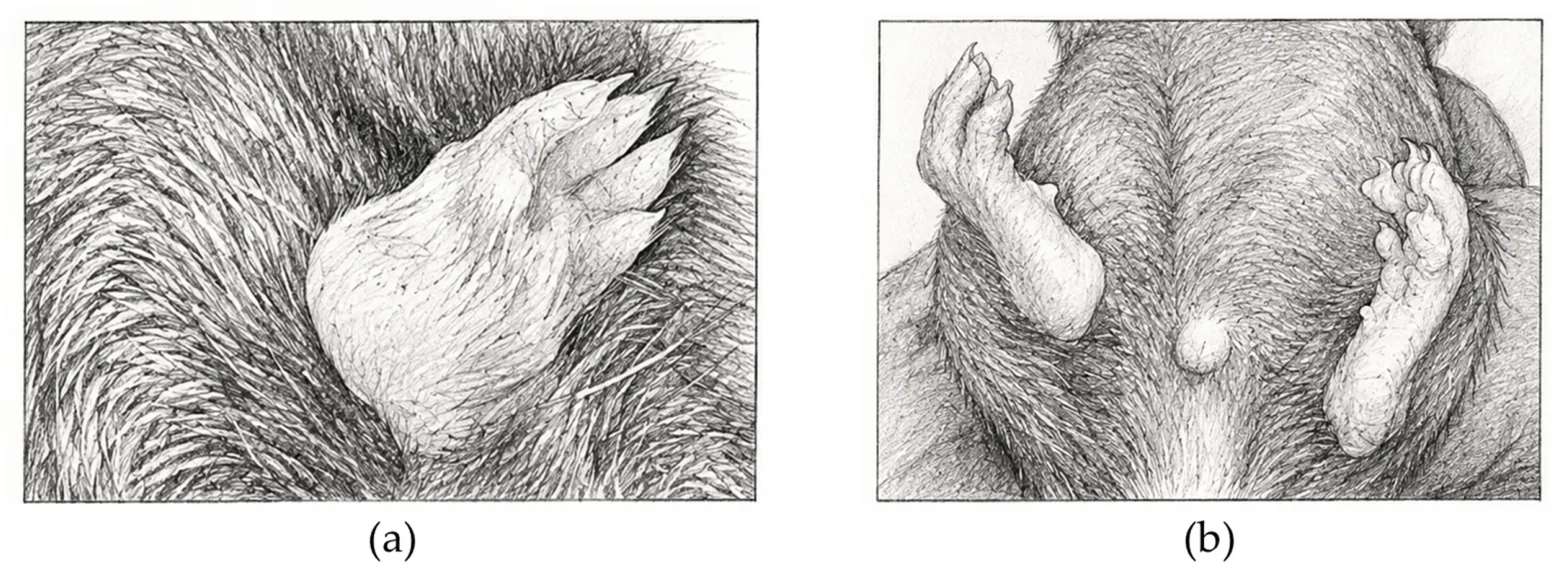
Representative Sketches of (**a**) Forepaw and (**b**) Hindpaw Swelling in the Human TNF-transgenic (hTNF-Tg) arthritis mouse model. The animals show swollen paws associated with bone malformation.

**Table 1:** Comparative Overview of Major Murine Models Used in Rheumatoid Arthritis Research.

Model	Primary Mechanism	Major Immune Components	Key RA Features Recapitulated	Major Advantages	Important Limitations
CIA	Active immunization with type II collagen induces autoimmune arthritis	CII-specific T cells, B cells, autoantibodies, macrophages, neutrophils	Synovitis, pannus formation, autoantibody production, cartilage destruction, bone erosion	Well-characterized; models initiation and progression of autoimmune arthritis; widely used for therapeutic testing	Strain/MHC-dependent; induced autoimmune response differs from human RA heterogeneity
CAIA	Passive transfer of anti-CII antibodies induces immune complex-mediated inflammation.	Fcγ receptors, complement, neutrophils, macrophages	Synovitis, inflammatory infiltration, cartilage damage, bone erosion	Rapid, synchronized disease onset; excellent for studying inflammatory effector pathways and drug efficacy	Does not model autoimmune initiation or adaptive immune priming
hTNF-Tg	Constitutive expression of human TNF-α drives chronic inflammation	TNF-dependent macrophage activation, fibroblast activation, osteoclastogenesis	Chronic synovitis, pannus formation, cartilage degradation, bone erosion	Strong translational relevance for TNF biology; responsive to anti-TNF therapies	Artificial cytokine overexpression; limited modeling of autoimmunity initiation
SKG	ZAP-70 mutation creates autoreactive T cells; zymosan provides environmental trigger	Th17 cells, IL-17, innate immune receptors, macrophages, dendritic cells	Symmetric polyarthritis, synovitis, cartilage destruction, bone erosion	Models gene–environment interaction; valuable for studying Th17-driven autoimmune mechanisms	Disease variability; environmental trigger does not fully represent human RA complexity

**Table 2:** Summary of immune cells involved in CIA, CAIA, hTNF-Tg, and SKG mouse models of arthritis. Contribution scale: +++, substantial contribution; ++, moderate contribution; +, secondary contribution; +−++, Low to moderate contribution, with involvement varying by disease stage; ++−++, Moderate to high contribution, with involvement varying by disease stage.

Immune Cell	CIA	CAIA	hTNF-Tg	SKG
CD4^+^ T cells	+++ CII-specific T-cell responses; provide help for B-cell responses and contribute to joint inflammation	+ Limited contribution to disease initiation	+ Secondary contribution to TNF-driven inflammation	+++ Autoreactive CD4^+^ T cells initiate and sustain autoimmune inflammation
Th17 cells	+++ Promote inflammation and tissue injury through IL-17A	+ May contribute to inflammatory amplification	++ Can participate in downstream inflammatory responses	+++ Major T-cell population associated with IL-17A-mediated inflammation
Th1 cells	++ Contribute to cellular inflammatory responses; effects may vary with disease stage	+ Limited role in antibody-mediated disease induction	+ Secondary contribution	++ Contribute to inflammatory responses alongside Th17 cells
Treg cells	++ Modulate autoreactive immune responses	+ Limited role during antibody-induced disease initiation	+−++ Potential regulation of chronic inflammation	++ Regulation of autoreactive T-cell responses influences disease development
CD8^+^ T cells	++ Contribute to inflammatory and cytotoxic responses	+ Limited contribution to disease initiation	+ Secondary contribution	++ Contribute to inflammatory responses
B cells	+++ Produce anti-CII antibodies and participate in antigen presentation	+ Not required for initiation because antibodies are passively transferred	+ Limited contribution to TNF-driven disease	++ Contribute to humoral and antigen-presenting responses
Plasma cells	+++ Produce pathogenic anti-CII antibodies	+ Limited role in initiation because pathogenic antibodies are administered exogenously	+ Limited evidence for a primary role	++ Contribute to autoantibody production
Complement	++−++ Amplifies antibody/immune-complex-mediated inflammation	+++ Important mediator of anti-CII antibody-induced inflammation	+−++ May amplify inflammation but is not the primary initiating pathway	++ May contribute to inflammatory amplification
Neutrophils	+++ Joint recruitment and inflammatory tissue injury	+++ Major effector population recruited following antibody/FcγR and complement activation	++−++ Recruited during TNF-driven inflammation	+++ Contribute to acute joint inflammation and tissue injury
Macrophages	+++ Produce inflammatory mediators and promote synovitis and tissue damage	+++ Important FcγR-dependent inflammatory effector cells	+++ Major TNF-responsive inflammatory population	+++ Amplify T-cell/Th17-associated inflammation
Dendritic cells	++ Antigen presentation and T-cell priming	+−++ Contribute to inflammatory responses	++ Participate in inflammatory signaling	+++ Support activation of autoreactive T-cell responses
NK cells	++ Contribute to inflammatory cytokine responses	++ May contribute to innate inflammatory amplification	+ Secondary contribution	++ Participate in inflammatory immune responses
Mast cells	++ Contribute to inflammatory mediator release	++ May participate in antibody-mediated inflammation	+ Secondary contribution	++ Contribute to inflammatory mediator release
Osteoclasts	+++ Mediate inflammatory bone resorption	++ Contribute to antibody-associated bone damage	+++ TNF-associated osteoclast activation and bone erosion	+++ Contribute to inflammatory bone destruction

**Table 3:** Summary of inflammatory mediators involved in CIA, CAIA, hTNF-Tg, and SKG mouse models of arthritis.

Cytokine	CIA	CAIA	hTNF-Tg	SKG Arthritis
TNF-α	Major effector cytokine; drives synovitis, cartilage damage, bone erosion	Essential amplifier of antibody-mediated inflammation	Primary disease driver (overexpression)	Contributes to inflammation but not primary initiator
IL-1β	Critical for cartilage destruction and bone erosion	One of the dominant mediators downstream of immune complexes	Strong downstream mediator of TNF signaling	Promotes joint inflammation
IL-6	Promotes Th17 differentiation and chronic inflammation	Contributes to inflammatory amplification	Elevated downstream of TNF	Important for Th17 expansion and disease severity
IL-17A	Major pathogenic cytokine from Th17 cells	Low to moderate contribution	Secondary downstream mediator	Central pathogenic cytokine driving disease
IFN-γ	Modulates adaptive immunity; context-dependent (protective and pathogenic roles reported)	Minimal role	Low contribution	Variable; influences Th1/Th17 balance
GM-CSF	Promotes activation of myeloid cells and osteoclastogenesis	Supports neutrophil/macrophage activation	Important downstream inflammatory mediator	Critical effector cytokine from pathogenic T cells
IL-23	Maintains pathogenic Th17 cells	Limited role	Low to moderate	Essential for pathogenic Th17 responses
IL-12	Supports Th1 differentiation	Minor role	Low	Moderate role in T-cell polarization
IL-10	Regulatory; suppresses disease severity	Regulatory	Regulatory	Regulatory; deficiency worsens disease
TGF-β	Required for Th17 differentiation but also immunoregulatory	Minor role	Low	Important in Th17 differentiation with IL-6
RANKL	Drives osteoclast differentiation and bone erosion	Important in erosive disease	High due to TNF-driven osteoclastogenesis	Promotes bone destruction
CXCL1/CXCL2	Recruit neutrophils	Major neutrophil chemoattractants	Elevated	Elevated
CCL2 (MCP-1)	Recruits monocytes/macrophages	Important in effector phase	Elevated	Elevated

## Data Availability

No new data were created or analyzed in this study. Data sharing is not applicable to this article.

## Abbreviations

The following abbreviations are used in this manuscript:

RA	Rheumatoid Arthritis
CIA	Collagen-Induced Arthritis
CAIA	Collagen Antibody-Induced Arthritis
DMARDs	Disease-Modifying Antirheumatic Drugs
TNF-α	Tumor Necrosis Factor-alpha
hTNF-Tg	Human Tumor Necrosis Factor Transgenic
CII	Type II Collagen
FcγR	Fc Gamma Receptor
MHC	Major Histocompatibility Complex
LPS	Lipopolysaccharide
ZAP-70	Zeta-Chain-Associated Protein Kinase 70
SPF	Specific Pathogen-Free
CLEC7A	C-type Lectin Domain family 7 member A
TLR2	Toll-like Receptor 2
Syk	Spleen Tyrosine Kinase
NF-kB	Nuclear Factor Kappa B
IL	Interleukin
GM-CSF	Granulocyte–Macrophage Colony-Stimulating Factor
W163C	Tryptophan(W) replaces with Cystein (C) at position 163 in the carboxyl-terminal SH2 domain of ZAP-70
JAK	Janus Kinase
Th17	T helper 17
TCR	T-cell receptor
